# Using concept mapping to develop a human rights based indicator framework to assess country efforts to strengthen rehabilitation provision and policy: the Rehabilitation System Diagnosis and Dialogue framework (RESYST)

**DOI:** 10.1186/s12992-018-0410-5

**Published:** 2018-10-01

**Authors:** Dimitrios Skempes, John Melvin, Per von Groote, Gerold Stucki, Jerome Bickenbach

**Affiliations:** 1 Department of Health Sciences and Health Policy, Center for Rehabilitation in Global Health Systems, University of Lucerne, Frohburgstrasse 3, P.O. Box 4466, CH-6002 Lucerne, Switzerland; 2 Swiss Paraplegic Research (SPF), Guido A. Zaech Institute, CH-6207 Nottwil, Switzerland; 3 Department of Rehabilitation Medicine, Thomas Jefferson University, 25 S. Ninth Street, Philadelphia, PA 19107 USA; 4 Institute of Social and Preventive Medicine (ISPM), University of Bern, Mittelstrasse 43, CH-3012 Bern, Switzerland

**Keywords:** Health services for persons with disability, Rehabilitation, Health systems, Indicators, Convention on the Rights of Persons with Disabilities, Right to health, Monitoring, Accountability

## Abstract

**Background:**

Rehabilitation is crucial for the realization of the right to health and a proper concern of global health. Yet, reliable information to guide rehabilitation service planning is unavailable in many countries in part due to the lack of appropriate indicators. To ensure universal health coverage and meet the central imperative of “leaving no one behind” countries must be able to assess key aspects of rehabilitation policy and provision and monitor how they have discharged their human rights responsibilities towards those most disadvantaged, including people with disability. This article describes the process of developing an expert guided indicator framework to assess governments’ efforts and progress in strengthening rehabilitation in line with the Convention on the Rights of Persons with Disabilities.

**Methods:**

A systems methodology - concept mapping - was used to capture, aggregate and confirm the knowledge of diverse stakeholders on measures thought to be useful for monitoring the implementation of the Convention with respect to health related rehabilitation. Fifty-six individuals generated a list of 107 indicators through online brainstorming which were subsequently sorted by 37 experts from the original panel into non overlapping categories. Forty-one participants rated the indicators for importance and feasibility. Multivariate statistical techniques where used to explore patterns and themes in the data and create the indicators’ organizing framework which was verified and interpreted by a select number of participants.

**Results:**

A concept map of 11 clusters of indicators emerged from the analysis grouped into three broader themes: Governance and Leadership (3 clusters); Service Delivery, Financing and Oversight (6 clusters); and Human Resources (2 clusters). The indicator framework was comprehensive and well aligned with the Convention. On average, there was a moderately positive correlation between importance and feasibility of the indicators (*r* = .58) with experts prioritizing the indicators contained in the clusters of the Governance and Leadership domain. Two of the most important indicators arose from the Service Delivery, Financing and Oversight domain and reflect the need to monitor unmet needs and barriers in access to rehabilitation. In total, 59 indicators achieved above average score for importance and comprised the two–tiered priority set of indicators.

**Conclusion:**

Concept mapping was successful in generating a shared model that enables a system’s view of the most critical legal, policy and programmatic factors that must be addressed when assessing country efforts to reform, upscale and improve rehabilitation services. The Rehabilitation Systems Diagnosis and Dialogue framework provides a data driven basis for the development of standardized data collection tools to facilitate comparative analysis of rehabilitation systems. Despite agreement on the importance and feasibility of 59 indicators, further research is needed to appraise the applicability and utility of the indicators and secure a realistic assessment of rehabilitation systems.

**Electronic supplementary material:**

The online version of this article (10.1186/s12992-018-0410-5) contains supplementary material, which is available to authorized users.

## Background

Rehabilitation is an integral component of the therapeutic continuum that every health system must strengthen on the path to universal health coverage (UHC) [1]. Today there is a nearly universal consensus that access to health related rehabilitation (HRR) is indispensable for the realization of the right to health and a fundamental precondition for the societal inclusion of persons living with disability. This consensus is reflected in the United Nations Convention on the Rights of Persons with Disabilities (CRPD) [2]– the most comprehensive binding legal instrument on the protection of the rights of persons with disabilities - which has been ratified by 87% of the world’s nations and requires signatories to organize, strengthen and extend HRR programmes to ensure equitable and timely access to quality services [3]. In the 10 years that have transpired since the proclamation of the CRPD, recognition of the importance of rehabilitation has grown considerably [4–13]. Acknowledgment is also increasing that alignment of rehabilitation policies, systems and services with the standards of the CRPD is necessary if we are to ensure a stronger, effective and more holistic response to the diverse rehabilitation needs of the ever expanding population living with disability [14–20]. To ensure countries meet their international legal obligations and achieve meaningful progress towards UHC, the World Health Organization (WHO) has identified equity oriented monitoring and the development of indicators for persons with disabilities as a global priority [21], including indicators for rehabilitation [22].

Indicators, broadly speaking, are succinct measures that aim to provide the most comprehensive picture of the health system (or a subcomponent of it) possible with the least amount of unnecessary detail [23]. At the health system level, improving rehabilitation services requires robust and continuous monitoring with different types of indicators, including levels and distribution of rehabilitation workforce, service availability and quality, disease and disability prevalence/incidence and functional outcome indicators which can be derived from multiple sources. While these indicators focus primarily on inputs, outputs and outcomes, human rights indicators focus on health and service outcomes, but bring additional attention to equitable access to services as well as to the policies and practices of legal and administrative entities monitoring their efforts to create and sustain just social arrangements for the full realization of human rights [24]. Human rights indicators seek to addresses particularly the needs and rights of those made vulnerable by discrimination, lack of policy attention and socio-political power imbalances, such as persons with disabilities. In rehabilitation for example, rights based indicators would not be only concerned with measuring improvements in functioning and community integration, but seek to provide information about the following important aspects in the process of organizing and delivering rehabilitation services in health systems:

- Are human rights respected, protected and fulfilled in service delivery?
- Are the key principles of human rights met in policy formulation and implementation– Is the right to access to rehabilitation realized equally for all, with the active participation of service users, effective accountability mechanisms and without discrimination?
- What barriers do persons with disabilities experience in accessing appropriate rehabilitation? Are efforts being made to progressively remove those barriers?
- Is there an enabling environment for the implementation of human rights - Is the social, institutional, legal, organizational and economic environment conducive to the realization of the right to health and rehabilitation?

The development and use of such indicators is particularly pertinent to heath systems, because some of the most chronic and entrenched deficiencies in rehabilitation services are a direct consequence of the denial of human rights of persons with disabilities and result from frailties in the way health policies are formulated and implemented and laws are developed and enacted [4].

It is generally agreed that indicators must be embedded in a clear and robust underlying conceptual framework that specifies system domains or target areas for improvement. However, in the literature there is a gap on indicator frameworks that can help obtain a comprehensive picture of the weaknesses in rehabilitation service delivery and identify systemic failures in policy development and CRPD implementation. Approaches to monitoring rehabilitation systems and services vary considerably and there is very little agreement over the domains and elements of a global indicator framework. Without a strong empirical understanding of what such a framework should comprise of– including its key domains, subdomains and measures – progress with the implementation of the CRPD and other widely agreed recommendations on rehabilitation will be difficult.

Specifically, much of the existing research has focused on the development of clinical governance measures for various disease groups for the purpose of assessing improvements in quality of HRR [25–33]. Along with quality of care indicators, community based rehabilitation (CBR) indicators have generated interest [34]. These indicators take the form of surveys to capture user’s experiences of a range of broad measures across several domains of social policy [35]. Important though these indicators are, it is not clear that in the aggregate they constitute an adequate account of the human rights obligations of States in relation to HRR [3] as they tend to overemphasize individual outcomes rather than health policy structures and organizational processes. Moreover, their strict focus on community oriented inclusive development measures makes them, to a significant degree, inappropriate for monitoring the HRR sector, not only in affluent nations, where the application of WHO’s community based rehabilitation framework is very limited or problematic [36], but also in less developed countries [37]. In fact, researchers have started experimenting with frameworks developed in other health areas in an effort to assess the capacity of health system to deliver HRR in low-income countries [38], which indicates the growing need for a health sector specific framework to monitor rehabilitation services.

Three recent systematic efforts have developed approaches to monitor the status, performance and quality of HRR services respectively: the International Classification of Service Organization in Rehabilitation (ICSO-R) [39], the Rehabilitative Care System and Capacity Planning Evaluation Frameworks [40, 41], and the Rehabilitation Management System (RMS) [42, 43]. While these initiatives have resulted in interesting monitoring frameworks and their use has yielded important and valuable insights for health policy, they do not strictly make use of human rights indicators but mainly focus on describing and assessing the technical performance of service delivery. Additionally they did not seem to aim for a level of comprehensiveness necessary to assess the legal, governance and strategic planning aspects of rehabilitation. A concrete focus on these aspects is essential to monitor States progress in enhancing rehabilitation system performance in compliance with international human rights law standards (e.g., prohibition of disability discrimination in health insurance, service user participation in service design, existence of national action plan on rehabilitation, promulgation and enforcement of accessibility standards for healthcare facilities, etc.). Moreover, the methods by which these frameworks were developed were not clearly presented and do not seem to follow a rigorous process. On the other hand, empirical approaches to understand and assess how health related rights are respected in disability and rehabilitation policies [44] confine themselves to the analysis of States commitments as expressed in policy documents and miss important aspects of actual service performance which limits their breadth and relevance for monitoring system level issues of HRR.

To help countries meet their obligations under the CRPD and achieve greater and more equitable improvements in service delivery, there is an urgent need to develop a conceptually robust indicator framework for HRR. To be useful both for human rights accountability and monitoring States’ efforts to strengthen and scale up HRR services within the context of Sustainable Development Goals (SDG), such a framework must faithfully account for a wide range of legal, policy and programmatic factors that influence equitable access to rehabilitation and assistive technologies. To achieve this, the indicators included in the framework should be informed by key provisions of the CRPD pertaining to rehabilitation to drive the collection of data consistently with international human rights standards [45]. Furthermore, because the organization of rehabilitation services varies significantly across healthcare systems, there is a need for expert consensus regarding standardized approaches to assessing HRR at country level.

In light of these considerations, we conducted a study to develop an expert-informed indicator framework for assessing country efforts to strengthen rehabilitation through implementation of the CRPD. Specifically the objectives of this study were: (a) to elicit and synthesize the knowledge of experts on indicators that would be useful for monitoring the implementation of the HRR aspects of the CRPD; (b) to integrate and confirm this knowledge through feedback to develop a shared conceptual model for rehabilitation sector assessment; and (c) to prioritize a set of important indicators to guide future evaluation and research.

In meeting the objectives above, we have adopted the following definitions:

### Health related rehabilitation

In an effort to acknowledge the critical connections between rehabilitation services and the health system we use the term “health related rehabilitation” in the same way as in the context of Article 25 of the CRPD [2] to denote individualized, outcome focused healthcare services. Specifically we adopted the definition proposed by Meyer and colleagues [46], which takes into account HRR at the micro, meso and macro level with an emphasis on organizational and system characteristics of HRR services. Ideally, these services consist of scientifically sound and evidence based diagnostic, treatment and therapeutic activities as well as other interventions that are regulated and organized by the health sector. They are typically delivered in an organisational setting by a multidisciplinary team of properly trained and certified professionals (or a single therapist when appropriate).

### Persons with disability

Because rehabilitation, by definition, targets persons whose functions are limited as a result of illness, injury or chronic disease, it is very rare for a HRR intervention or service to benefit only persons with long term impairments and disabilities. Thus an approach to defining “persons with disabilities” for the purpose of monitoring HRR should be broad enough to account for the variety of disease groups that may benefit from HRR. To maintain consistency with the CRPD, “persons with disability” are understood in this research “to include those women, men, girls and boys with long-term physical, mental, intellectual or sensory impairments which, in interaction with various barriers, may hinder their full and effective participation in society on an equal basis with others” [2]. However, the characterization of “persons with disabilities” contained in the CRPD does not restrict coverage to particular persons; rather, it identifies persons with long-term physical, mental, intellectual and sensory disabilities as the main class to be protected. The reference to “include” in Article 1 of the Convention could therefore extend its application to all persons with functional limitations, i.e., those with short-term impairments or persons who are perceived to be part of such groups such as people with controllable non communicable diseases and episodic disabilities[47, 48]. This view is also reflected in WHO’s Global Action Plan on Disability 2014–2021 “Better health for all people with disabilities” which is informed by the principles of the CRPD and UHC, among others [5]. As with the earlier World Report on Disability, the WHO global action plan directs governments’ attention to the health and rehabilitation needs of people with disabilities asserting that this concrete focus will lead to faster and more sustainable improvements in rehabilitation services and thus bring about larger benefits for all people who may need rehabilitation in the context of UHC. By the same token, this research and its resulting product will be relevant to all those who are traditionally understood as disabled and face restrictions in everyday participation but also to clinical populations who experience limitations in functioning.

## Methods

### Study design

In accordance with our study protocol [49] we used an innovative form of structured conceptualization, colloquially referred to as *group concept mapping,* to develop the indicator framework which we call the Rehabilitation Systems Diagnosis and Dialogue (RESYST) framework*.* Group Concept mapping (GCM) is a mixed method that integrates sound qualitative group procedures (brainstorming, categorizing ideas, and assigning value ratings) with multivariate statistical analyses to help a group describe their ideas on the topic of interest and represent these ideas visually through a series of related maps [50]. It is considered a well-established method in public health research [51, 52] that has been applied successfully in the past to develop measurement and evaluation frameworks and tools [53]. Acknowledging the utility and value of GCM as a participatory approach that democratizes the process of knowledge co-production [54, 55], experts in rehabilitation research have recommended GCM as an appropriate technique of concept elicitation that helps generate useful insights from diverse stakeholders to guide the development of standardized measurement tools [56]. In addition, as a mixed method, GCM deploys advanced statistical techniques that allow data from multiple sources to be combined and analysed to produce a mental model that reflects the collective thinking of participants [57]. This feature makes GCM particularly useful given the objective of the study to develop a consensus understanding of rights based monitoring of rehabilitation policies and programmes.

### Procedures

GCM typically involves the following stages: preparation, including participants identification and recruitment; idea generation; structuring of ideas into piles of similar themes and rating them against predefined criteria; data representation; and group interpretation. All data collection and analysis procedures were performed with Concept Systems Global Max© [58] and were accomplished in the period between March 2015 – March 2017. The steps taken to achieve the goals of our study are explained in the text below:

#### Preparation

The interdisciplinary nature of the research question required us to ensure that a variety of perspectives were represented in the sample. Therefore the research team modified a previously developed recruitment strategy [59] to facilitate the identification of a multidisciplinary group of knowledgeable individuals engaged in disability-inclusive health development in various capacities: policy decision making, research and education, advocacy and professional practice. Initially it was estimated that a minimum number of 150 individuals should be identified and invited to participate in the structured activities to ensure an adequate number of participants complete the sorting task and thus guarantee the reliability of the framework [49].

English speaking individuals were *purposively* selected and included in the stakeholders pool using the following criteria: (a) participants should possess knowledge and experience in various domains of rehabilitation systems development such as disability law and policy, disability statistics and information, rehabilitation services and policy, CBR, assistive technologies, clinical rehabilitation, and professional training and education as demonstrated by peer reviewed scientific publications; (b) participants should possess knowledge of the human rights approach to disability as demonstrated through participation in technical expert panels issuing rights based, disability policy recommendations. Participants were also judged as suitable for inclusion if they were recommended by other participants or research team members as appropriate stakeholders or content experts. Stakeholders were identified by reviewing the following sources: lists of attendees at technical meetings and conferences organized by international agencies (UN, World Bank); lists of authors and contributors of major WHO publications pertinent to rehabilitation; lists of experts appointed by WHO to provide advice in the development of guidelines and standards for rehabilitation and assistive technologies; author lists of articles published in high impact peer reviewed journals; and the authors’ personal contacts lists. In addition we screened the list of international nongovernemental and professional organizations in official relation with WHO [60] and searched the websites of those whose activities are relevant to HRR to identify experts that would be interested in contributing to this project. This process resulted in the identification of 221 key stakeholders.

Although the indicator generation phase of the study was arranged with a larger number of participants to capture diversity of opinions (heterogeneity sampling), the grouping of indicators into distinct categories required a more homogenous group of participants, one that “share the same conditions and has the basic organization to discuss and validate individual members’ experiences collectively, notwithstanding their internal diversity, and to take action based on this discussion” ([61]p. 41). Therefore a criterion sampling technique was applied to select experts from the initial stakeholders pool. Individuals were judged as suitable experts if they satisfied one of following additional criteria: (1) relevant science experience/knowledge of monitoring and evaluation as demonstrated by peer reviewed publications; (2) ability to influence monitoring practices and tool application as demonstrated by affiliation with an organization involved in monitoring and evaluation of rehabilitation services, such as intergovernmental health agencies, professional and advocacy organizations, and research institutions, or participation in international standard setting processes, such as policy guidelines development.

Six international experts were selected from the study sample and invited to serve on a steering committee to guide the overall implementation of the GCM study. Members of the committee helped recruit additional participants into the study and could partake in any or all steps of the concept mapping process. Subsequently, the research team prepared a background document, with input from members of the Advisory Committee, containing information on the purpose and methodology of the project, definitions of key terms and a summary of the human rights obligations of States in relation to rehabilitation based on previously published evidence [3]. Drawing upon Article 31 of the CRPD, the team expanded the background document to include a set of core principles for the identification of indicators along with a preliminary list of 127 candidate indicators compiled from a focused review of the literature and the authors’ notes from discussions with experts. Finally, to facilitate the collection of meaningful input, the study team, with guidance from the Advisory Committee, developed and pilot tested a focus question to which stakeholders responded: “*A specific indicator that would help assess progress in the implementation of the health related rehabilitation aspects of the Convention on the Rights of Persons with Disabilities is*…”.

#### Idea generation

Participant stakeholders were asked to provide input on specific indicators relevant for monitoring the implementation of the HRR aspects of the CRPD using the above prompt as the focus for the structured responses. Stakeholders were contacted via e-mail and provided with a web address for a project-specific, asynchronous platform through which they could submit their ideas anonymously online. The project specific website contained a link to the background document which participants could access and download. Participants were given 3 weeks to respond. At the conclusion of the brainstorming session the first author (DS) synthesized the responses in preparation for the subsequent phases. Initially, doubled barrelled statements were split into their components and the set was reduced by removing duplicate statements or statements containing very similar ideas. Subsequently the statements were reviewed independently by two reviewers (DS, PvG) for relevance to the focus question, clarity and comprehensibility. Any disagreements that arose were resolved by consensus or with a third reviewer (JB). The consolidated brainstormed statements set of ideas was then randomized and uploaded onto the project website for the structuring phase.

#### Structuring

Structuring of ideas consisted of participants sorting and rating the synthesized set of indicators electronically. Sorting activity consisted of participants individually grouping the indicators into conceptually similar categories or piles and providing labels to each pile they created to reflect the indicators within. The instructions stated that each indicator belonged to only one pile and that participants should not group all indicators into one pile or create a “miscellaneous” pile. Participants were also asked to provide answers to a brief socio-demographic questionnaire. Concurrently, all participants were invited to rate each indicator on a 4 point scale for importance and potential feasibility of obtaining or collecting the data related to the indicator, compared to all other indicators within the set (1 = relatively unimportant/not at all feasible, 4 = very important/feasible).

#### Representation

The concept mapping analysis was based on the aggregation of individual sort data [62]. The first step involved the creation of a *N* x *N* binary square similarity matrix to represent each individual’s sort data. Rows and columns of the matrix correspond to the indicators generated in the brainstorming phase. The values within the cells of the matrix represented whether (1) or not (0) the participant sorted indicators into the same pile. The individual matrices of all sorters were then aggregated to create a single similarity matrix which showed how the entire group sorted the indicators. Non metric multidimensional scaling (MDS) of the aggregated similarity matrix enabled the creation of a two-dimensional visual representation of the indicators (point map) where the relative distance between them indicates the degree of their relative similarity [63, 64].

Subsequently, hierarchical cluster analysis partitioned the point map into non overlapping clusters in a way such that indicators that were in adjacent areas of the map were placed in the same cluster [65]. The output of the cluster analysis was a cluster map which revealed how the indicators (represented by numbered points) were categorized into higher order themes. Because cluster analysis is a heuristic tool there is no standard mathematical criterion for selecting the final number of clusters. The research team followed the procedures described by Kane and Trochim [50] to determine the most meaningfully interpretable representation of the indicators in the conceptual model. Agglomerative nesting was used to examine a range of cluster solutions in order to identify a cluster configuration where separation or merger of clusters adequately represented the data as organized by the participants. Bridging index, a measure of the degree to which an indicator was sorted by participants with other indicators in the vicinity, was generated to estimate the internal consistency of each cluster. Bridging values range from 0 to 1 with lower values indicating more conceptually robust clusters. Finally, measures of central tendency and measures of dispersion were computed to identify and describe patterns in participants rating data. Pearson product moment correlation (r) was calculated to estimate the degree of the overall agreement between respondents’ average cluster ratings on importance and feasibility as well as the degree of agreement between subgroups of respondents on the same variables (defined by geographic region, level of knowledge of the CRPD and stakeholder group). All analyses are considered to be exploratory.

#### Group interpretation

Interpretation of results involved receiving input from a convenience sample of participants during a half day meeting held in Nottwil, Switzerland. Seven individuals participated in a face to face meeting which was facilitated by a member of the research team. The interpretation group comprised of academic and clinical experts in rehabilitation medicine (*n* = 3), experts in disability law and policy (*n* = 2), an expert in vocational rehabilitation with lived experience of disability and a health policy scientist. At the interpretation session participants reviewed or modified as allowed the preliminary cluster solution, collectively assigned labels to each cluster and discussed the content of each cluster in light of the rating results. This information provided the basis for researchers and participants to co-finalize and interpret the concept map by identifying regions of thematically related clusters. All group decisions were made by consensus.

### Ethics review and approval

The Ethics Commission for Northwest and Central Switzerland considered ethical approval not necessary. All participants were assured prior to engaging in this study of data confidentiality, informed of the voluntary nature of their participation, and of their possibility to withdraw at any time.

## Results

### Participants

Fifty six individuals generated 275 initial ideas in response to the focus question requesting indicators that would be useful to monitor the HRR aspects of the CRPD. These ideas were consolidated in a final set of 107 unique statements by the research team (see Additional file 1). Subsequently, 44 completed the on-line sorting and/or rating tasks. Specifically, 37 participants completed the sorting task, 41 completed the importance rating and 39 the feasibility rating. Participant characteristics are presented in Table 1. Affiliations of individual experts who participated in the sorting and rating phase are shown in the Additional file 2.

**Table 1:** Participant characteristics

Participant characteristics	Sorting and rating (*N* = 44)
	Respondents n (%)
Stakeholder group	
Administrators and agency leaders	5 (12%)
Disabled people’s representatives, advocates and disability inclusive development practitioners involved in rehabilitation	5 (11%)
Rehabilitation professionals and/or academic researchers	34 (77%)
did not respond	0 (0%)
Primary area of expertise	
Clinical Functional Rehabilitation	8 (18%)
(physical, psychosocial or occupational)	
Community based rehabilitation	5 (12%)
Assistive technologies	2 (5%)
Rehabilitation services and policies	12 (27%)
Professional training and education	1 (2%)
Disability data and statistics	11 (25%)
Disability law and policy	5 (11%)
did not respond	0 (0%)
Years of professional experience	
< 5 years	2 (4%)
5–14 years	13 (30%)
> 15 years	29 (66%)
did not respond	0 (0%)
Knowledge of the CRPD	
Have never heard of it	0 (0%)
Fair	7 (16%)
Good	13 (29%)
Excellent	24 (55%)
did not respond	0 (0%)
Location	
Africa	4 (9%)
America	8 (18%)
South-East Asia	7 (16%)
Europe	18 (41%)
Eastern Mediterranean	1 (2%)
Western Pacific	6 (14%)
did not respond	0 (0%)

To ensure the reliability of data analysis concept mapping guidelines recommend a number of 10–40 participants to complete the sorting activity [50]. In this study, 74% of those who agreed to participate in the sorting phase completed the sorting activity (*N* = 37), thus falling within the recommended range. Although 41 participants have originally completed the ratings, importance and feasibility ratings of 4 and 3 individuals respectively were excluded from the final analysis because of extreme response patterns and missing data. Finally, of the seven individuals who took part in the group interpretation, four have completed both sorting and rating activities.

### Development of the Rehabilitation Systems Diagnosis and Dialogue framework (RESYST)

MDS analysis of the sort data produced a two-dimensional point map with a stress value of 0.269^1^ which was below the average stress value of 0.28 (SD=0.04, range: 0.17; 0.34) as estimated in a recent meta-analytic study of GCM projects [66]. Thus, the point map was considered as sufficiently reliable to proceed with further analyses. Application of hierarchical cluster analysis to the point map resulted in a preliminary framework solution of 11 clusters which was judged by the research team to provide sufficient detail and still yield substantially interpretable content within each cluster.

During the interpretation session, participants reviewed the 11 cluster solution and modified it as allowed to increase within cluster consistency of content and enhance the map’s overall interpretability. For example, the boundaries of the cluster labelled *Service Coverage, Utilization and Outcomes* were redrawn to include also Indicator #31 (see Additional file 1), which, initially belonged to the *Social Mobilization and Research* cluster, but was perceived by participants to be more conceptually related with service delivery outcomes. Similarly, the cluster *Workforce Development* was reshaped to include Indicator #1 (see Additional file 1) from the *Evidence informed and Rights based Programming* cluster, as participants felt it made more sense to be grouped with the indicators that pertain to workforce issues. It is important to note that these changes aimed at improving the overall interpretability of the map and did not alter the underlying data structure (point map) which reflects how participants sorted the indicators.

**Fig. 1:**
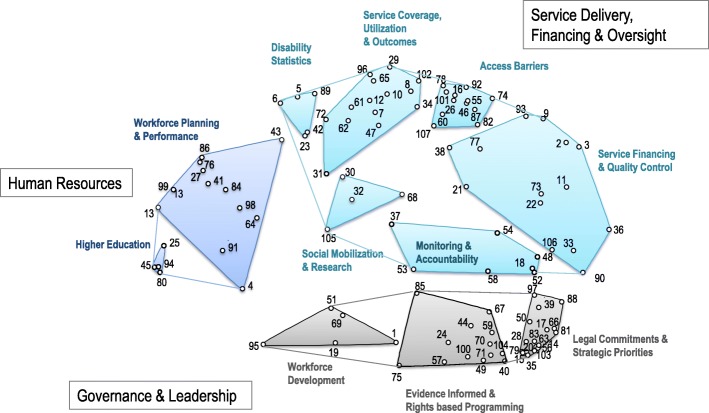
Concept map with 11 clusters of indicators illustrating three thematic regions. This figure presents the 11 clusters that emerged from the concept mapping process and their grouping into higher order themes and reflect expert consensus on the key domains and subdomains of the Rehabilitation Systems Diagnosis and Dialogue - RESYST framework. The emergent mental model is grounded on the 107 indicators that experts perceived as useful for monitoring the implementation of the CRPD in regards to HRR which constitute the key elements of the framework. The label assigned to each cluster reflect the common theme of its indicators. Numbers on the map correspond to the brainstormed indicators (see Additional file 1)

The final conceptual structure that emerged from the analysis and interpretation of the sorting information organized the 107 indicators into 11 non overlapping clusters as shown in Fig. 1. Experts who participated in the interpretation session confirmed the appropriateness of the cluster labels. Each cluster is described below in descending order of average importance. The full list of indicators within each cluster of the RESYST framework is presented in the Additional file 1.

*The Legal Commitments and Strategic Priorities* cluster contained the most indicators (17), which reflects the fact that the ideas included in this cluster were most frequently brainstormed by the participants. It was also the most dense construct that emerged from the analysis with high internal coherence as evidenced by a bridging value of .09. This cluster encompassed measures that capture rules, norms and processes at the level of the government that aim to protect human rights (Indicators #20, #56, #79, #103) and promote the right to health and rehabilitation (Indicators #14, #35, #83). It also includes indicators that assess the existence of laws and standards to ensure legal access to comprehensive and effective quality rehabilitation care (Indicators #63, #81, #88) and qualitative measures that examine efforts to mainstream rehabilitation in strategic health care planning (Indicators #17, #39, #50, #66).

*Monitoring and Accountability* contained 7 indicators focusing on the existence of information systems to track rehabilitation resources (Indicators #37, #52) and the availability and use of intelligence to ensure evidence informed management and public accountability (Indicators #48, #58). Some indicators however were thought to be conceptually related with ideas contained in adjacent clusters (Indicators #53, #54) as shown by their mid-range bringing values (see Additional file 1).

*Evidence informed and Rights based Programming* included 13 statements emphasizing the need for operational planning tools (Indicators #70, #104) as means to promote evidence based decision making and as well as critical policy structures and mechanisms (Indicators #40, #44, #57, #67, #71, #100) that States must put in place to strengthen rights based, inclusive rehabilitation policy making and ensure the implementation of the CRPD. This cluster had a bridging value of .25 which indicates the high degree of consensus among experts on the relatedness of the items in the cluster and was internally stable and coherent.

The *Workforce Development* cluster encompassed 5 indicators related to measures taken by governments to ensure the sustainability and awareness of the rehabilitation workforce of the rights of persons with disabilities. This cluster had a bridging value of .76, suggesting that the items contained within the cluster may be good matches with adjacent clusters. It should be noted however that the space between this cluster and the clusters of the Human Resource domain suggests that participants perceived the items contained in these clusters to be conceptually delineated.

*Access Barriers* contained 12 indicators capturing structural (Indicators #60, #78, #92), organizational (Indicators #16, #46, #55, #74, #87, #101) and financial (Indicators #46, #82) barriers to access to HRR and assistive devices. This cluster included the indicator rated highest for importance across the entire set of 107 indicators (Self-reported barriers to access to medical rehabilitation [Indicator #46], see Additional file 1).

The cluster *Service Coverage, Utilization and Outcomes* was comprised of 14 indicators and had a comparatively low bridging value of .37. This cluster contained the indicator “Unmet needs for medical rehabilitation” (Indicator #65, see Additional file 1) which was rated second highest for importance across the whole set of indicators.

The *Service Financing and Quality Control* cluster is composed of 14 indicators which, as the name of the cluster implies, participants perceived as useful to assess the allocation and investment of financial resources to improve access to rehabilitation services (Indicators #11, #36, #73, #90, #106), as well as measures to enhance the coordination (Indicator #77) and quality of rehabilitation care (Indicators #9, #38), including through accreditation (Indicator #93) and inspection of health facilities’ compliance with human rights (Indicators #2, #3). Evidence on the existence and content of a nationally determined set of essential rehabilitative services was the most important indicator in this cluster (Indicator #33). This cluster occupied the largest area in the concept map which suggests that the indicators contained therein were more loosely related and were highly likely to be sorted with other indicators in the map.

The cluster labelled *Higher Education* was the smallest cluster in the concept map and included 5 indicators addressing issues of academic training of rehabilitation professionals. Although this cluster had a modest bridging value of .66, its small size indicates that participants perceived its content as being conceptually distinct from other clusters, hence its position in the bottom left edge of the map.

The adjacent cluster *Workforce Planning and Performance* is comprised of 12 indicators that reflect the need for, and the measures though which, governments and other interested organizations can assess the availability (Indicators #27, #41, #43, #86, #99) of the rehabilitation workforce. It also includes a range of patient reported experience measures (PREM) (Indicators #4, #13, #64, #84, #91) thought to be related to rehabilitation providers and workforce performance. This cluster had the highest bridging value across all clusters (.77) which suggests that the ideas contained within the cluster are thematically more broad and diverse in comparison with indicators contained in other clusters on the map.

The relatively small cluster labelled *Disability Statistics* contained 5 indicators, including prevalence and incidence (Indicator #6, #89) as well as measures thought to be useful for public health (Indicator #23) and disability policy strategies (Indicator #5, #42). The importance of this cluster was relatively low. However, the standard deviation of 1.06 shows that individual responses, on average, were a little over 1 point away from the mean, which was the highest found across all clusters, suggesting that there was a great deal of variation in individual importance ratings within this cluster.

Finally, the *Social Mobilization and Research* cluster contained indicators that address capture efforts to promote empowerment (Indicator #30) a and culture of inclusiveness among health system stakeholders (Indicator #68) as well as efforts to promote rehabilitation research (Indicator #32, #105). The central location of this cluster indicates that the items contained in the cluster were highly likely to be sorted with items in other clusters in the map.

### Prioritization of indicators

**Fig. 2:**
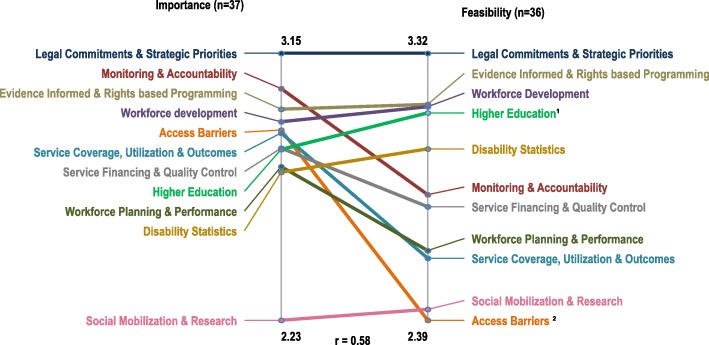
Pattern match display comparing importance versus feasibility ratings by cluster. Importance and feasibility rating scales are represented by the two vertical lines. Clusters are positioned on each line in descending order of importance and feasibility respectively. Rating values refer to average cluster ratings derived from average indicator ratings from within each cluster. Overall, the correlation between the ratings for importance and feasibility was moderately positive (*r* = .58). This indicates that participants perceptions of the importance are well aligned with their perceptions of feasibility. The degree of slope of the lines connecting cluster ratings on the left (Importance) to same ratings on the right (Feasibility) illustrates this alignment. For example, there was a great deal of correspondence between importance and feasibility to implement the indicators contained in the clusters of the Governance and Leadership domain. Also, all participants agreed on the relative low importance and feasibility of the *Social Mobilisation and Research* cluster. Conversely, the majority of clusters of Service Delivery, Financing and Oversight were, on average, perceived as relatively less important and less feasible to implement with the exception of the cluster *Monitoring and Accountability* which was ranked second highest for importance. Indicators of barriers to access to rehabilitation were rated almost as important as *Service Coverage, Utilization and Outcomes* but hardest to implement. *Service Financing and Quality Control* and *Higher Education* clusters were perceived equally important, although indicators in the former were thought to be more difficult to implement. ^1^*p* < 0.02, ^2^*p* < 0.005

Rating information captured how important and feasible experts who took part in this study perceived the indicators to be. Indicator rating scores have been averaged per cluster (Additional file 1) and are also presented in the form of a ladder graph in Fig. 2. The overall mean rating for importance per cluster ranged from 3.15 (SD = .87, 95% CI: 3.08; 3.22) for the cluster *Legal Commitments and Strategic Priorities* to 2.23 (SD = .57, 95% CI: 2.09; 2.37) for the cluster *Social Mobilization and Research*. On average, the *Legal Commitments and Strategic Priorities,* which was perceived as the most important, achieved the highest rating score for feasibility whereas indicators contained in the cluster *Access Barriers* were on average perceived as the least feasible to implement.

Intergroup comparisons of importance and feasibility ratings were conducted to identify patterns of convergence and divergence in the views of participants with differing sociodemographic characteristics. The results showed that there was no significant variation in the responses and most participants followed similar patterns in prioritizing the indicators. For example, the results showed that there was a high degree of consensus between the group of “Rehabilitation professionals and/or academic researchers” and participants belonging to all other groups on both importance (*r* = .86, *p* < 0.01) and feasibility (*r* = .95, *p* < 0.01) ratings. Similar patterns were found in additional analyses with intergroup correlations ranging from .69 (*p* < 0.05) to .98 (*p* < 0.01).

**Fig. 3:**
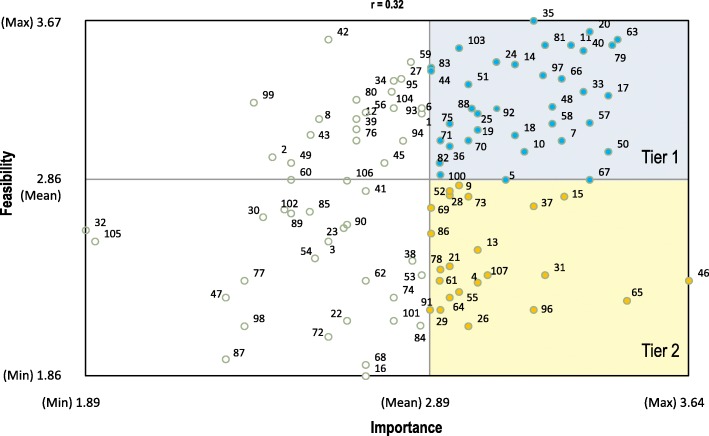
Bivariate plot mapping importance versus feasibility ratings for 107 indicators. The box plot is divided into quadrants on the basis of the overall mean value for each of the rating variables. Numbered points correspond to the indicators enumerated in the Additional file 1. Blue and yellow points indicate the 59 indicators that achieved above average score for importance and comprise the priority set of rehabilitation indicators. On average, the indicators in the upper right quadrant (blue shaded area/points) achieved above average score for both importance and feasibility and represent the implementation priority set of indicators (Tier 1) whereas indicators in the lower right quadrant (yellow shaded area/points) received below average score for feasibility and comprise the development priority set (Tier 2)

Figure 3 presents a bivariate plot mapping average importance and feasibility ratings for 107 indicators. This plot helped participants during the interpretation session examine the relationship between importance and feasibility at the item level and derive a two-tier set of priority indicators for further field testing.

Overall, 59 brainstormed statements were rated above average for importance and comprised the priority set of human rights indicators for rehabilitation (Table 2). Of those 36 indicators achieved above average score for both importance and feasibility and were perceived by participants as having higher potential for success in monitoring the implementation of the CRPD (Tier 1: Implementation priority, see Fig. 3). Two thirds (*n* = 24) of the indicators in Tier 1 arose from the Governance and Leadership domain with the majority (*n* = 13) belonging to the *Legal Commitments and Strategic Priorities* cluster. Conversely, 23 indicators obtained above average rating for importance but below average for feasibility (Tier 2: Development priority set, see Fig. 3) which, according to the participants, represent areas where investments need to be made to systematize and improve the collection of data so as to enable the effective review and assessment of the rehabilitation sector in the future. Interestingly, the priority indicators of the *Workforce Planning and Performance* cluster (see Table 2) were seen as less feasible to implement in comparison with other indicators within this cluster.

**Table 2:** Priority set of 59 indicators rated above average for relative importance, arranged by cluster in descending order of importance

No^a^	Indicators per cluster	Importance	
		Mean	SSD
	**Legal Commitments and Strategic Priorities**	**3.15** ^b^	**−0.87**
**63** ^c^	The State has a law to ensure universal access to comprehensive rehabilitative care and assistive products for all (yes/no).	3.43	−0.77
**79**	State law explicitly prohibits discrimination in health insurance on the ground of disability or other pre-existing condition (yes/no).	3.42	−0.77
**17**	National health or disability strategy addresses priority health related rehabilitation issues (yes/no). Describe and specify. Timeframe and coverage.	3.41	−0.76
**50**	Evidence documenting (a) establishment of an operational, budgeted, multi sectoral national rehabilitation action plan aligned with WHO international and or regional action plans, (b) target setting process, (c) implementation activities, (d) monitoring and evaluation plan.	3.41	−0.72
**20**	Constitutional guarantees to disability equality - The State takes at least one approach to disability equality and non-discrimination (yes/no).	3.35	−0.92
15	The concept of disability used in health laws, policies, programmes and regulations and in the collection of relevant statistical data is in line with the human rights approach to disability and the protection of the rights of all persons with disabilities regardless of impairment (yes/no)	3.28	−0.88
**66**	National disaster preparedness and relief plans are inclusive of health related rehabilitation (yes/no).	3.27	−0.8
**81**	Legally binding national accessibility standards/guidelines established and documented (yes/no). Year of adoption.	3.22	−0.93
**97**	Existence of an Operational Unit, Branch or Dept. in the Ministry of Health (or other Ministry) with responsibility for rehabilitation services/ assistive technologies policy development, implementation, monitoring and evaluation (yes/no). Jurisdiction and scope.	3.22	−0.79
**35**	Status of ratification of international human rights treaties recognizing the right to health and their optional protocols.	3.19	−0.88
**14**	Date of entry into force and coverage of domestic legislation for the implementation of the right to health of persons with disability, including legislation on rehabilitation care.	3.14	−0.79
**88**	Existence of government approved evidence based guidelines for the rehabilitation of a wide range of disabling conditions through a multidisciplinary team approach (yes/no).	3.03	−0.83
**103**	Legislative provision prohibiting compulsory medical treatment and experimentation (yes/no).	2.97	−1.07
28	State regulations require healthcare providers to implement policies, procedures and/or protocols for partnering with patients, carers and consumers in: (i) Strategic and operational/services planning (yes/no) (ii) Decision-making about safety and quality initiatives (yes/no) (iii) Quality improvement activities (yes/no).	2.95	−0.78
**83**	Date of entry into force and coverage of the right to health of persons with disability in the constitution or other form of superior law.	2.89	−0.99
	**Monitoring and Accountability**	**3.03**	**−0.83**
**58**	Existence of a national set of relevant indicators with targets and annual reporting to inform annual rehabilitation sector reviews and other planning cycles (yes/no).	3.24	−0.89
**48**	The State has conducted an overall assessment of the performance of the rehabilitation care system in the last 5 years (yes/no).	3.24	−0.8
37	Availability of an integrated information system on the health-related rehabilitation workforce, providing periodically updated data on the size, type, geographical distribution, competencies and skill mix of the national stock of workers.	3.19	−0.78
**18**	Rehabilitation service delivery regulations, quality specifications and professional standards are established and documented (yes/no).Year of last update.	3.14	−0.75
52	Existence of a unified accounting system to track allocation of funds to health related rehabilitation services integrated within the overall health expenditure tracking system (yes/no).	2.95	−0.88
	**Evidence Informed and Rights Based Programming**	**2.96**	**−0.86**
**67**	Evidence on the existence of formal collaboration between (a) the department/agency responsible for rehabilitation and (b) the department/agency responsible for: (i) employment, (ii) education, (iii) welfare (iii) CRPD implementation.	3.35	−0.63
**57**	State has established inclusive procedures or mechanisms for consultation with disabled people’s organizations at national, sub-national and local levels (yes/no).	3.35	−0.72
**40**	Existence of national multi-sectoral commission, agency or mechanism for the coordination of disability policy and the implementation of the CRPD (yes/no). Scope & functions.	3.33	−0.83
**24**	Charter of patient rights published and available in accessible formats (yes/no).	3.08	−0.89
**70**	The State has a systematic plan and coordinating unit for acquiring and using rehabilitation research information and for sharing and transferring knowledge (yes/no).	3	−0.78
**75**	Existence of a participatory forum and disability inclusive process to coordinate the setting of national rehabilitation research priorities (yes/no).	2.95	−0.94
**71**	The State has a budgeted plan to raise awareness about disability issues among health professionals which involves persons with disabilities and their representative organizations (yes/no). Timeframe and coverage.	2.92	−0.8
**100**	Existence of accessible pre-judicial mechanisms to lodge complaints alleging breach of obligations connected to the right to health. Jurisdiction and scope.	2.92	−0.83
**44**	Existence of a government website which meets the ISO/IEC 40500:2012 standards of accessibility for web content with latest report and data about rehabilitation services available to the general public (yes/no).	2.89	−0.97
	**Workforce Development**	**2.91**	**−0.87**
**19**	Existence of disability human rights education as an element of the accreditation standards used at the national level in the field of rehabilitation.	3.03	−0.76
**51**	Availability of ethical standards of care for rehabilitation physicians and allied health professionals (yes/no).	3	−0.78
69	Existence of human resources for health unit that is responsible for developing and monitoring policies and plans on rehabilitation workforce and negotiating intersectoral relationships with other line ministries and stakeholders (yes/no).	2.89	−0.94
	**Access Barriers**	**2.89**	**−0.86**
46	Barriers in access to medical rehabilitation (%) - Reported number of persons with disabilities not having access to medical rehabilitation services due to transportation barriers, physical/geographical access barriers, waiting time, lack of information; lack of time; inadequate skills of service provider; cost or other.	3.64	−0.59
**92**	Percentage (%) of health facilities providing medical rehabilitation services.	3.08	−0.76
107	Needs for assistive products met - Reported number of persons with disability using an assistive product that fits their functional needs.	3.06	−0.75
26	Inequality in access to rehabilitation - Absolute difference in unmet needs for rehabilitation between people with and without clinical impairments/disabilities (trends).	3	−0.94
55	Timely access to rehabilitation 2 - Time (median waiting time in days) between: (i) acute hospital admission until referral for rehabilitation, (ii) referral until assessment, (iii) acceptance by post-acute rehabilitation care and ready for transfer until admission.	2.97	−0.93
78	Proportion of the population living within four hours travel to a rehabilitation/assistive technology service (Allows for visiting a service within a day.)	2.92	−0.98
**82**	Assistive technology affordability - Percentage (%) of the per capita GDP or income required to purchase a wheelchair (average price).	2.92	−0.87
	**Service Coverage, Utilization and Outcomes**	**2.87**	**−0.85**
65	Unmet needs for medical rehabilitation - Reported number of persons with disability that needed medical rehabilitation services or assistive devices in the last 12 months and did not get the services they need, stratified by age, income, geographic region and educational level	3.46	−0.77
**7**	Number of Community Based Rehabilitation providers/population ratio (per 100,000)	3.27	−0.69
31	Financial barriers in access to assistive technology - Reported number of persons with disability who didn’t get their prescribed assistive devices because of their cost	3.22	−0.8
96	Financial barriers in access to rehabilitation - Reported number of persons with disability that have forgone prescribed rehabilitation treatment due to financial reasons in the last 12 months, disaggregated by income level, sex and age	3.19	−0.84
**10**	Number of multidisciplinary rehabilitation programmes per 1,000,000 - (e.g., cardiac, cancer, stroke, spinal cord injury, paediatric rehabilitation programmes).	3.16	−0.76
61	Patient status at discharge - National average percentage (%) of rehabilitation inpatients with improved function scores at discharge (compared with scores measured at admission).	2.92	−0.97
29	Proportion of persons with disability living in complex emergency environments that can access comprehensive rehabilitation services	2.92	−0.89
	**Service Financing and Quality Control**	**2.82**	**−0.85**
**33**	A comprehensive array of medical rehabilitation services is enlisted in the State’s essential health benefits package including for the purpose of maintaining current levels of functioning (yes/no). Describe and specify.	3.33	−0.83
**11**	Percentage (%) of WHO recommended priority assistive products included in the national assistive products list for procurement and reimbursement.	3.3	−0.78
73	Expenditure trends on (i) rehabilitation care (inpatient, outpatient and community based) as % of government health expenditure (ii) assistive products as % of government health expenditure.	3	−0.82
9	Percentage (%) of health facilities/units offering medical rehabilitation with established quality improvement teams, by facility type.	2.97	−0.9
21	Evidence (including of qualitative nature) of gender sensitiveness of rehabilitation services.	2.95	−0.85
**36**	The State subsidizes disabled people’s travel costs to access rehabilitation services that are not available near their place of residence.	2.95	−0.85
	**Higher Education**	**2.82**	**−0.85**
**25**	Training in physical medicine and rehabilitation available for doctors. This refers to a residency programme in Physical Medicine and Rehabilitation (PMR) or specialist certification in PRM which is recognized by the medical council or the equivalent licensing body of the country (yes/no).	3.03	−0.83
	**Workforce Planning and Performance**	**2.76**	**−0.85**
13	Self-perceived community integration – Percentage (%) of survey respondents with disability who would rate their level of community integration as “7”out of “10” or higher.	3.03	−0.96
4	Percentage (%) of persons with disability reporting having personally felt discriminated against or harassed during rehabilitation within the last 12 months on the basis of a ground of discrimination prohibited under international human rights law (compared to people without disability).	3.03	−0.8
64	Percentage (%) of persons with disability that feel they have received sufficient information and been sufficiently involved in making decisions about their rehabilitation treatment compared to people without disability	2.95	−0.91
86	Rehabilitation workforce density by occupation/specialization and activity level.	2.89	−0.84
91	Percentage (%) of rehabilitation service users who said they have been sufficiently involved in decisions about their care as much as they wanted to be.	2.89	−0.82
	**Disability Statistics**	**2.74**	**−1.06**
**5**	Return to work rates - Average national percentage (%) of vocational rehabilitation clients of working age who are engaged in sustainable employment 3–6 months after closure and were employed before entering vocational rehabilitation.	3.11	−1.02

^a^Number corresponds to the number that was randomly assigned to the indicator after the brainstorming phase

^b^Cluster rating scores are based on the mean rating for all indicators within the cluster

^c^Bolded numbers indicate inclusion of the indicator in the implementation priority set (Tier 1)

### Mapping the RESYST onto the CRPD

The 107 indicators of the RESYST framework were mapped onto the CRPD. Table 3 shows the relevance of the indicators generated from concept mapping for monitoring human rights norms and standards that are implicated in HRR and recognized in the CRPD. All 107 address directly or indirectly fundamental political and social rights in relation to HRR. The majority of the indicators cover the right to health and rehabilitation as expressed in Articles 25 and 26 whereas a large number of indicators capture States efforts to promote the implementation of the CRPD by raising awareness of disability rights among rehabilitation workers and promoting their professional development.

**Table 3:** Relevance of the 107 indicators for monitoring human rights implicated in health related rehabilitation planning and programming specified in the CRPD

			Rehabilitation Systems Diagnosis and Dialogue - RESYST framework											
		Framework Domains	Governance & Leadership			Service Delivery, Financing & Oversight						Human Resources		
		Cluster labels	Legal Commitments & Strategic Priorities	Evidence Informed & Rights based Programming	Workforce Development	Monitoring & Accountability	Service Financing & Quality Control	Access Barriers	Service Coverage, Utilization & Outcomes	Disability Statistics	Social Mobilization & Research	Workforce Planning & Performance	Higher Education	CRPD Provisions
Rehabilitation in the CRPD	Human rights & Fundamental freedoms	Equality before & under the law / Non discrimination	20, 14, 83											4 (1), 4 (1) (b), 5 (2), 6 (1), 7 (1), 25, 25 (e), 25 (f)
		Freedom of Expression and Opinion & Right to Information		24, 44										4 (1) (h), 21
		Right to Respect Physical and Mental Integrity										64		17, 25 (d)
		Freedom from Torture, or other cruel, Inhuman, or degrading treatment of punishment	103											15 (1), (15 (2)
		Freedom from Exploitation, Violence and Abuse					3							16 (3)
		Right to Privacy		59			2							22
		Right to Health	79, 63, 35, 17, 66, 50, 97, 88	40, 49, 67	1, 51	18	77, 90, 21, 93, 33, 73, 9, 11, 36	101, 78, 55, 46, 26, 87, 16, 107, 92, 74, 60, 82	8, 102, 34, 61, 65, 47, 12, 62, 72, 10, 29, 7, 96, 31			43, 76, 86, 84, 4		11, 25, 25 (a), 25 (b), 25 (c), 26, 26 (2), 26 (3)
		Right to Participation in the Conduct of Public Affairs & Right to Active, Informed and Meaningful Participation	28	57, 75			38				30	91		4(3), 29
		Right to Inclusion and Independent Living								5		13		19
	Standards & Enabling measures	Professional Training & Awareness Raising		71	19, 95	54					68	41, 27, 99, 98	94, 45, 80, 25	4 (1) (i), 20 (c), 25 (d), 26 (2)
		Statistics and Data Collection	15		69	52, 58, 48, 37	22			89, 23, 42, 6				31 (1), 31 (2), 31 (3), 33 (1), 33 (2), 33 (3)
		Accessibility	56, 81											9 (1), 9 (2)
		Personal Mobility						82						20 (b)
		Access to Justice		100		53								13
		International Assistance and Cooperation (incl. Research & Knowledge Promotion)	39	104, 70, 85			106				32, 105			4 (1) (g), 32 (a), 32 (b)

Numbers in the cells correspond to the brainstormed indicators (see Additional file 1)

## Discussion

In this study, we used GCM to synthesize the perspectives of rehabilitation stakeholders to systematically identify important and feasible indicators to assess country efforts to strengthen rehabilitation systems and services in line with CRPD requirements. The final framework included measures that address the most important legal, policy and programmatic factors that have been shown to facilitate or constrain access to rehabilitation services such as laws and regulations, strategic planning, accountability mechanisms, service delivery, financing and human resources [4]. The findings of the present study provide the preliminary evidence base for future efforts to assess governments’ response to population rehabilitation needs in line with their international human rights obligations.

The three groupings of clusters in the concept map resemble to key building blocks of WHO’s Framework for Action on Strengthening Health Systems [67] which provides empirical validation for viewing the rehabilitation sector as a subsystem of the overall health system. Health systems have been described as complex adaptive systems [68], meaning that they are constantly changing as they are susceptible to internal and external influences, and are composed of various subsystems. In the context of rehabilitation, it has been argued that decision making teams and service organizations operate in a chaotic environment and thus constitute self-defined, unpredictable subsystems of broader healthcare systems characterized also by complexity and interconnectedness whereby components of these subsystem affect each other [69–72]. In our conceptual model, the words in the cluster labels represent inputs, outputs, initial and/or final outcomes but also processes, flows, controls and context across the system strengthening chain. Each of these words triggers a response by the others, e.g. legal commitments dictate the establishment of national priorities, optimal workforce planning drives better performance, effective coverage leads to enhanced utilization. Specifically, the *Legal Commitments and Strategic Priorities* cluster underlines the obligation of States to incorporate the key provisions of the CRPD in domestic legislation [73](Indicators #103, #20, #79, #14, #15, #56, #81) as a crucial means in realizing the right to health of persons with disabilities [74] and strengthening health systems [75, 76] and suggests that national priorities in the context of SDGs (Indicators #17, #66, #39) be informed by the international human rights standards, a view that is shared widely among leading commentators and global health experts [77–79]. A similar interpretation can occur across other clusters of indicators allowing an in depth examination of systemic issues pertaining to rehabilitation service delivery, financing and workforce planning.

Analysis of the spatial features of the concept map reveals interesting findings about the interrelationship of indicators and clusters and may enhance our understanding of the interdependent and complex nature of rehabilitation systems organization. The Governance and Leadership domain and clusters of the Service Delivery, Financing and Oversight are both seen as being closely related to *Monitoring and Accountability*. This appears to affirm the paramount role of health information systems and accountability structures for both effective service delivery and good governance [80, 81]. For example it is recommended that national action plans on sectoral health issues include a monitoring and evaluation plan [82] (Indicator #50, *Legal Commitments and Strategic Priorities*). But more importantly, effective implementation of such plans require a set of national indicators and benchmarks to facilitate annual reporting of progress against agreed objectives (Indicator #58, *Monitoring and Accountability*) [83]. Additionally, the capacity to collect and process rehabilitation expenditure data through sophisticated accounting infrastructure (Indicator #52, *Monitoring and Accountability*) is essential for monitoring expenditure trends (Indicator #73, *Service Financing and Quality Control*), including development aid flows (Indicator #106, *Service Financing and Quality Control*), and prioritizing investments in assistive technologies (Indicator #90, *Service Financing and Quality Control*).

These findings point to the complex interrelationship of the fundamental inter-organizational norms, inputs and functionalities that underpin effective and responsive rehabilitation service systems and suggest that challenges in the implementation of the CRPD need to be considered holistically as an integrated network of multiple agents (people, organizations, resources, rules and norms) and perspectives. They thus offers a novel focus on the intersection between human rights standards and the core functions of the health system as drivers of variation in access to HRR. In this respect, our framework makes a unique contribution to global health and disability policy as it combines a normative, human rights lens with a systems approach to strengthening rehabilitation that has been missing from the current literature. As shown in Table 3, the RESYST bridges the monitoring and analysis of the human rights implicated in rehabilitation with an assessment of the broader system within which CRPD implementation efforts are being realized which helps formulate well defined and appropriate boundaries for the implementation of the right to access rehabilitation. This dynamic combination offers a powerful means to re-focus stakeholder actions and government priorities from the often paralytic analysis and repetition of policy recommendations to “strengthen rehabilitation” to effective strategies for accountability and system change.

While many of the results of this project reinforce those from previous studies suggesting indicators to monitor important aspects of rehabilitation services and policy such as admission and discharge barriers to medical rehabilitation [84], multi-level barriers in access to community based rehabilitation [85], patient satisfaction [86, 87], rehabilitation workforce density [88] etc., there were several new findings. Indicators capturing the legal and regulatory landscape of rehabilitation services, which ranked highly in both importance and feasibility, were not mentioned in previous studies, except with reference to the ratification of human right treaties [89, 90] and the recognition of disabled people’s right to health in national constitutions [91]. The existence of a national strategy and action plan for rehabilitation (#17, #50), the establishment of administrative structures and mechanisms for cross sectoral coordination of policy (#40, #67, #97), the existence of accessible mechanisms to ensure and promote service users participation in rehabilitation policy decision making, service design and monitoring (#28, #57, #75) are all new indicators that reflect key ingredients for the success of efforts to implement the CRPD and strengthen rehabilitation service systems [17]. Further, indicators on the capacity of the rehabilitation system to generate and use strategic intelligence to empower citizens to claim their rights (#48, #53) and inform policy decision making at various levels (#18, #52, #58, #37) appear in the priority set of indicators. These indicators are foundational to realizing the right to access rehabilitation as they provide policy signals regarding efforts to ensure sound decision making and public accountability, which are core principles of good governance in HRR [20].

Additionally, assistive technology indicators have received little attention in previous studies. In our study these included measures of affordability (#31, #82), service and financial coverage (#11, #65, #107), geographic access (#60, #78), patient experience measures (#84) as well as indicators that capture States’ policy efforts to promote the availability and use of assistive technologies(#39, #49, #63, #73, #90, #97). These indicators reflect the strong link between health systems, assistive devices and human rights [18, 92–94] and therefore highlight the need to consider assistive devices in future service and policy audits especially in the context of SDG monitoring [95].

Finally, participants in this study identified important affirmative measures such as the adoption of legislation for the protection and implementation of health related rights of persons with disabilities (#14, #59, #83, #104), the existence of comprehensive anti-discrimination legislation (#20, #79), the promulgation (#81) and enforcement of accessibility standards (#56) and raising awareness of disability human rights issues among health professionals (#71) as facilitators of access to HRR and thus key elements of a comprehensive indicator framework. Moreover the group identified measures of absolute inequality in access to rehabilitation between people with and without disability as highly important (#26). These results show that participants in our study were acutely aware of the importance of human rights for achieving sustainable and equitable progress towards UHC and cognisant of internationally accepted approaches to equity oriented monitoring [96].

At the cluster level, participants prioritized the indicators contained in the clusters *Legal Commitments and Strategic Priorities*, *Evidence Informed and Rights based Programming* and *Workforce Development* over all other indicators. Our findings reiterate findings in a recent Delphi study embedded within a realist review of governance related factors influencing the implementation of the CRPD [20]. Taken together, these findings indicate that governance indicators are central to the evaluation of rehabilitation services as they extend monitoring and analysis beyond the confines of clinical services and provide crucial evidence on progress towards creating and sustaining an enabling political and institutional environment for the realization of health related rights [17, 97].

Reinforcing results from previous studies [98, 99], priorities at the individual item level revolved around the need to identify barriers persons with disabilities experience in accessing rehabilitation services and to record unmet needs for rehabilitation. This information can help design targeted and effective strategies to enhance equity and responsiveness of the health system [100, 101] and reflects the obligation of States under Article 31 of the CRPD to collect data on barriers persons with disabilities experience in the enjoyment of their human rights [3].

What most experts agreed on was that there are important patient reported experience measures (PREM) addressing aspects such as patient dignity (#4), autonomy (#91), communication and access to information (#64) and support (#84) that need to be incorporated in some way as part of monitoring the performance of the rehabilitation professionals. Together with patient reported outcome measures which are used to monitor the progress of a health condition or whether a treatment has been effective by comparing results over time, PREM represent key outcomes of the performance of service providers [102] allowing for evaluation of services based on users experience which helps clinical leaders and policy planners understand failures that have led to unsatisfactory performance. The sorting of PREM with workforce indicators generates a new insight about how performance of rehabilitation providers can be assessed in the future. Specifically, the *Workforce Planning and Performance* cluster highlights a role for professionals that extends beyond the confines of disability management and positions them as negotiators and facilitators of human rights in rehabilitation care processes whereas the high importance rating assigned to PREM reiterates their significance as relevant and appropriate metrics of providers’ performance. These findings echo the views of experts who argue that inclusion of PREM in future review and assessments in neglected clinical areas (e.g., long term care, mental health) is essential because they reflect directly the voice of people with chronic conditions and carry, therefore, considerable potential to support improvements in patient centred care and enhance the quality of interactions between patients and providers [103].

### Implications and applications

The framework described in this paper has important policy implications. Firstly, the RESYST and its content constitute useful tools for the successful implementation of the WHO’s Global Action Plan on Disability, under which governments are responsible to “provide health sector support for monitoring and evaluating the implementation of health policies to ensure compliance with the provisions of the Convention on the Rights of Persons with Disabilities” [5][p.10] and to “undertake situation analysis to inform policies and planning” [5][p.17] for rehabilitation. Also, the RESYST provides the preliminary evidence base to support the implementation of WHO’s recommendation “to assess existing policies, systems, services and regulatory mechanisms, identifying gaps and priorities to improve provision” [4][p.122] of rehabilitation. The clusters and indicators offer a data driven basis for Ministries of Health to develop sector specific reporting tools, including country templates, that can guide the collection and analysis of pertinent information and provide a rapid indication of progress towards the implementation of the Action Plan and other widely agreed policy recommendations. Such structured templates and instruments have been developed and applied successfully in the context of mental health [104–106] and it is believed that replication of such an approach will impact positively the rehabilitation sector. Additionally, the indicators have the potential to inform the development of standardized data collection tools and resources by public health analysts and researchers, such as structured questionnaires, surveys and key informant interview guides and thus contribute to the harmonization of data collection practices in rehabilitation services and policy research. Populating the indicators with reliable and comparable data at regular intervals in the future may enable the comparative and longitudinal benchmarking of rehabilitation services and help decision makers draw more meaningful conclusions based on sophisticated country level analyses of the rehabilitation sector.

### Study limitations

There were methodological issues that may have impacted on our results and lessons learned will help adjust the design of similar studies in the future. Despite the implementation of evidence based measures to increase participation in our study (personalized email invitations, and frequent personalized reminders) participation rates were lower than the average concept-mapping project [66], for both importance and feasibility rating, meaning that our results may be prone to self-selection bias. Although the relatively low number of raters maybe a weakness, experience from the implementation of the GCM methodology suggests that the number of generated statements, the number of clusters and most importantly the number of sorters in our study were well within the limits of what is perceived as a standard to obtain reliable results. [66] Also, as is the case with all studies that employ non probability sampling techniques, the specific results of this study may have limited sample-to-population generalizability.

This research had a strong focus on the rehabilitation related dimensions of the right to health of persons with disabilities. We have therefore adopted a practical definition for HRR to facilitate a common understanding of the programmes and services that stakeholders think of as “health related rehabilitation”. This definition, along with evidence generated from a previous legal analysis of States obligations under the CRPD, helped participants identify potential measures that address several factors that impact on access to services and the enjoyment of disabled people’s right to health. This emphasis on the health care aspects of rehabilitation may have biased our selection of potential eligible participants in favour of those who are more active in the health domain. This has resulted in an overrepresentation of rehabilitation professionals and disability researchers in the group of sorters and raters. Additionally, given the general scepticism over contemporary approaches and definitions of rehabilitation by the disability community, and the subsequent lack of trust in rehabilitation research endeavours, it is possible that the emphasis on HRR has deterred some representatives of people with disabilities from participating in the sorting and rating of indicators. However our decision to concentrate on these aspects is justified by the widespread and often insurmountable barriers people with disabilities confront in accessing rehabilitation treatment services. It could be argued that an approach that would place a greater focus on social aspects of rehabilitation, especially CBR, may have created a more enabling environment and provided greater opportunities for persons with disabilities - especially those from low and middle income countries - to express their voice on this important topic and may have allowed to capture a small but significant segment of rehabilitation service systems in low income countries that is disproportionately focused on community inclusive development. Given however the expressed concerns over the ability of CBR as a strategy to bring broader systemic changes in line with the CRPD and remove barriers in access to services [107], it is doubtful whether this would be the right choice. Even so, it would have resulted in an entirely different set of indicators that would not have been bias free either.

Notably, experience from research shows that the issue of participation of service users or community members in the development of measures and indicators is highly controversial (as is the definition of stakeholders in community based research) as there are no specific guidelines regarding participation rates and the optimal balance between stakeholders groups [108, 109]. This lack of guidance partly explains the low to moderate levels of participation of patients and service users in community engaged concept mapping research on health systems, where the majority of studies examined in systematic review appeared to adopt a consultative rather a collaborative approach to community participation [55]. Informed by a human rights approach to disability, our study set a goal to create conditions that would allow bi-directional input and collaboration with persons with disabilities who have traditionally been neglected in the discussions about accountability and monitoring. [49] We paid particular attention to achieving a balance in the recruitment of both advisory committee members and individual participants to avoid creating hierarchies of power that would undermine the contributions of persons with disabilities by extending an invitation to the same number of individuals that were believed to belong to the same stakeholder group. Despite these efforts, several exogenous factors have impacted on participation rates. In addition to the lack of willingness discussed above, other commonly reported factors that may explain the low participation rates of persons with disabilities and policy makers include, among others, the labor intensive process of data collection, the high cognitive load induced by the high number of indicators and the lack of time [55]. Future research with larger sample sizes, including oversampling for specific stakeholder groups (policy makers and consumers) and a combination of web based and face to face data collection is recommended to counterbalance these limitations.

Interpretation of the conceptual map took place with a convenience sample of experts who participated in the sorting phase coming mainly from high-income countries. The lack of involvement of participants from low- and middle-income countries in the group interpretation may have introduced a culture bias as it is possible that the results would have evolved differently if their insights on the grouping of clusters into thematically related domains have been incorporated into the final model. Discussion of the cluster map and the indicator set with a larger number of participants - especially policy decision makers and service users –is recommended as part of future local adaptation and/or validation processes, as this may enhance their relevance for implementation as well as the sense of ownership of the results of the monitoring activity.

Although the framework incorporates a large number of factors previously found to be important in assessing progress in the implementation of the HRR aspects of the CRPD, it is possible that some indicators that are standardly used in rehabilitation service evaluations and quality assessments may have been missed. This was expected as the emphasis of this research was on human rights indicators intended to complement, rather than replace existing performance measures. It is likely that with further research, including testing and validation of the framework considering local context and stakeholder priorities, additional indicators may be included in the framework.

### Relationship with other frameworks

The RESYST presents both similarities and differences with the EQUIFRAME, a policy analysis framework that has been previously developed to facilitate the examination of the compatibility of health policies with human right guarantees for vulnerable populations [110], including in rehabilitation [44]. Given the both frameworks used human treaties as the normative basis for their development, it is not surprising that many of our indicators, especially indicators of governance, reflect core concepts featured in the EQUIFRAME. Unlike, however, EQUIFRAME which has a broader focus on health policies for marginalized populations, our framework focused particularly on HRR for people with disabilities. As a sector-specific framework, the RESYST is more extensive and more comprehensive: it encompasses measures that capture all aspects of the rehabilitation related dimensions of the CRPD, including quantitative indicators of service availability, service utilization and outcomes. Our framework also makes a distinction between CBR and HRR. Methodologically, the process of development of the RESYST was statistically more robust and resulted in a mental model in the form of a visual map that reflects the complexity and systemic nature of rehabilitation service organization noted also by others [71, 72].

### Recommendations

It will be important to test the validity and practical utility of the indicators in different contexts, assess the methodological constraints in data collection as well as the organizational costs and benefits associated with the use of the proposed indicators. This will require the drafting of indicator specification sheets (containing definition, rationale, method of calculation, interpretation and limitations for each indicator) and the development of multi-item assessment instruments to facilitate the collection, verification and analysis of information. The use of empirically developed and field tested indicators will provide opportunities to appraise rehabilitation policies and compare service organization across countries and thus move the scientific evidence base on comparative rehabilitation systems research forward. The practical insights offered by this study are both timely and strategically relevant as leading health agencies and professional organizations strive to integrate rehabilitation in health systems through capacity building and assessment initiatives. It is therefore recommended that health agencies, especially the WHO, professional organizations and international research consortia use the RESYST framework as an evidence source in future projects aiming to develop service monitoring and capacity assessment tools as well as to stimulate debate on methodological issues pertaining to the construction of system level indicators for rehabilitation.

## Conclusion

To our knowledge, this is the first systematic attempt to conceptualize the constituent domains and elements of a system level framework that details what, beyond traditional clinical outcomes or quality indicators, should be monitored to enable health programme planners implement evidence informed strategies to shape a more inclusive and pragmatic response to population rehabilitation needs as well as introduce and implement disability rights compliant policy reforms. As a conceptual device, the RESYST framework enables practitioners, researchers and advocates derive a complex understanding of the issues that must be considered in comprehensive rights based analyses and service audits. As a systems methodology, GCM was used to visualize and simplify the representation of multiple correlated legal, policy and programmatic variables that influence the implementation of the right to access to rehabilitation and helped make the connections between rehabilitation services and health systems more explicit. As a group decision tool, GCM allowed the collective thinking and priorities of a select group of experts and global scientists to surface which can more meaningfully direct efforts and inform future assessment of rehabilitation systems and policies.

The results reported here contribute to expanding the relatively limited evidence base of rehabilitation systems research and thus building stakeholders’ capacity for monitoring and evaluation. Future research should build on the experience of this study aiming at empirically testing the framework and the proposed measures and adapting it to local circumstances. Implementation of the RESYST after proper validation may help governments, and those seeking to support them, strengthen policy surveillance to gain a clearer and more comprehensive picture of the main weaknesses in rehabilitation services and align national strategies with obligations and commitments on disability rights and inclusion, thus leading to better and more equitable outcomes for all.

## Additional files

Additional file 1: Indicators per cluster. Rights based indicators for rehabilitation contributed by stakeholder-participants with Bridging Index and rating scores for relative importance and feasibility to implement, arranged by cluster in descending order of importance. (DOCX 37 kb)

Additional file 2: Stakeholder panel membership. Organizational affiliations of participants in the sorting and rating phase. (DOCX 77 kb)

## Abbreviations

CBR: Community Based Rehabilitation
CRPD: Convention on the Rights of Persons with Disabilities
GCM: Group Concept Mapping
HRR: Health Related Rehabilitation
MDS: Multidimensional Scaling
PREM: Patient Reported Experience Measures
RESYST: Rehabilitation System Diagnosis and Dialogue
SDG: Sustainable Development Goals
UN: United Nations
WHO: World Health Organization

## Footnotes

1 The stress score is a statistic that is standardly reported in MDS analyses and serves as an indicator of the goodness of fit between actual sort data and the point map’s configuration. Stress varies between 0 to 1 with lower scores indicating better fit.

## References

[1] Healthy systems for universal health coverage – a joint vision for healthy lives. 2017. https://www.uhc2030.org/fileadmin/uploads/uhc2030/Documents/About_UHC2030/mgt_arrangemts___docs/UHC2030_Official_documents/UHC2030_vision_paper_WEB2.pdf. Accessed 5 Aug 2018.

[2] Convention on the Rights of Persons with Disabilities. New York: United Nations, 2006. http://www.un.org/disabilities/documents/convention/convoptprot-e.pdf. Accessed 8 Aug 2018.

[3] Skempes D, Stucki G, Bickenbach J (2015). Health-related rehabilitation and human rights: analyzing states' obligations under the United Nations convention on the rights of persons with disabilities. Arch Phys Med Rehabil.

[4] World Health Organization and World Bank (2011). World report on disability.

[5] World Health Organization (2015). WHO global disability action plan 2014–2021: Better health for all people with disability.

[6] World Health Organization (2017). Rehabilitation in health systems.

[7] Stucki G, Bickenbach J, Gutenbrunner C, Melvin J (2018). Rehabilitation: The health strategy of the 21st century. Journal of Rehabilitation Medicine.

[8] White Book on Physical and Rehabilitation Medicine in Europe (2018). Chapter 2. Why rehabilitation is needed by individual and society. Eur J Phys Rehabil Med.

[9] Ng YS (2017). Rehabilitation medicine - the final frontier. Ann Acad Med Singap.

[10] Mitra R, Standaert CJ (2016). The value of physical medicine and rehabilitation in the new health care market. PM R.

[11] Grace SL, Turk-Adawi K, Santiago de Araujo Pio C, Alter DA (2016). Ensuring cardiac rehabilitation access for the majority of those in need: a call to action for Canada. Can J Cardiol.

[12] Charissa Levy SB, Emmi P (2016). Realizing the potential of rehabilitative Care for People with complex health conditions: the time is now. Health Care Q.

[13] Reinhardt JD, Li J, Gosney J (2011). Disability and health-related rehabilitation in international disaster relief. Glob Health Action.

[14] Stein MA, Stein PJS, Weiss D, Lang R (2009). Health care and the UN disability rights convention. Lancet.

[15] Bristo M, Blauwet CA, Frontera W (2014). The convention on the rights of persons with disabilities: what is at stake for physiatrists and the patients we serve. PM R.

[16] Giustini A, Von Groote PM, Christodoulou N, Michail X, Vanderstraeten G. Disability and human rights: the world report on disability as a unique opportunity to review and enrich European health policy. Eur J Phys Rehabil Med 2012;48(2):179–188. 22510679

[17] Skempes D, Bickenbach J (2015). Strengthening rehabilitation for people with disabilities: a human rights approach as the essential next step to accelerating global Progress. Am J Phys Med Rehabil.

[18] Borg J, Lindstrom A, Larsson S (2009). Assistive technology in developing countries: national and international responsibilities to implement the convention on the rights of persons with disabilities. Lancet.

[19] Hussey M, MacLachlan M, Mji G (2017). Barriers to the implementation of the health and rehabilitation articles of the United Nations convention on the rights of persons with disabilities in South Africa. Int J Health Policy Manag.

[20] McVeigh J, MacLachlan M, Gilmore B (2016). Promoting good policy for leadership and governance of health related rehabilitation: a realist synthesis. Glob Health.

[21] World Health Organization. Draft thirteenth general programme of work 2019–2023: Promote health, keep the world safe, serve the vulnerable. http://www.who.int/about/what-we-do/gpw13-expert-group/Draft-GPW13-Advance-Edited-5Jan2018.pdf. Accessed 8 Aug 2018.

[22] World Health Organization (2017). Health information systems and rehabilitation.

[23] NHS Institute for Innovation and Improvement (2008). The good indicators guide: Understanding how to use and choose indicators.

[24] United Nations Development Programme (2000). Using indicators for human rights accountability. Human Development Report 2000: Human rights and human development.

[25] Grace SL, Poirier P, Norris CM, Oakes GH, Somanader DS, Suskin N (2014). Pan-Canadian development of cardiac rehabilitation and secondary prevention quality indicators. Can J Cardiol.

[26] Zumsteg JM, Ennis SK, Jaffe KM, Mangione-Smith R, MacKenzie EJ, Rivara FP (2012). Quality of care indicators for the structure and organization of inpatient rehabilitation care of children with traumatic brain injury. Arch Phys Med Rehabil.

[27] Skjutar Å, Christensson K, Müllersdorf M (2009). Exploring indicators for pain rehabilitation: a Delphi study using a multidisciplinary expert panel. Musculoskeletal Care.

[28] Purvis T, Cadilhac D, Donnan G, Bernhardt J (2009). Systematic review of process indicators: including early rehabilitation interventions used to measure quality of acute stroke care. Int J Stroke.

[29] Farin E, Follert P, Gerdes N, Jäckel WH, Thalau J (2004). Quality assessment in rehabilitation centres: the indicator system ‘quality profile’. Disabil Rehabil.

[30] Ohtera S, Ueshima K, Nakayama T (2013). P275 Guideline-based quality indicators for cardiac rehabilitation of patients with ischemic heart disease in Japan: use of a modified Delphi technique for Indicator development. BMJ Quality & Safety.

[31] Guilcher SJT, Parsons D, Craven BC, Jaglal SB, Verrier M (2015). Developing quality of care indicators for patients with traumatic and non-traumatic spinal cord injury (SCI): a feasibility study using administrative health data. J Spinal Cord Med.

[32] Rivara FP, Ennis SK, Mangione-Smith R, EJ MK, Jaffe KM, The National Expert Panel for the Development of Pediatric Rehabilitation Quality Care I (2012). Quality of care indicators for the rehabilitation of children with traumatic brain injury. Arch Phys Med Rehabil.

[33] Killaspy H, White S, Wright C (2011). The development of the quality Indicator for rehabilitative care (QuIRC): a measure of best practice for facilities for people with longer term mental health problems. BMC Psychiatry.

[34] Lukersmith S, Hartley S, Kuipers P, Madden R, Llewellyn G, Dune T (2013). Community-based rehabilitation (CBR) monitoring and evaluation methods and tools: a literature review. Disabil Rehabil.

[35] World Health Organization (2015). Capturing the difference we make.

[36] la Cour K, Cutchin MP (2013). Developing community based rehabilitation for cancer survivors: organizing for coordination and coherence in practice. BMC Health Serv Res.

[37] M'kumbuzi V, Myezwa H, Myezwa H. Adaptation of the global frameworks for community based rehabilitation in southern Africa: a proof of concept. Rural Remote Health. 2017;17 http://www.rrh.org.au/articles/printviewnew.asp?ArticleID=3717. Accessed 17 Aug 2017. 10.22605/RRH3717 28835106

[38] Christian A, Bentley J, Aryeetey R, Ackuaku D, Mayer RS, Wegener S (2016). Assessment of rehabilitation capacity in Ghana. Disability CBR Inclusive Devel.

[39] Gutenbrunner C, Bickenbach J, Kiekens C (2015). ISPRM discussion paper: proposing dimensions for an international classification system for Service Organization in Health-related Rehabilitation. J Rehabil Med.

[40] Rehabilitative Care Alliance. Rehabilitative Care System Evaluation Framework. 2015. http://www.rehabcarealliance.ca/uploads/File/Final_Report_2013-15/CPSE/RCA_Evaluative_Framework_FINAL.pdf. Accessed 17 Aug 2017.

[41] Rehabilitative Care Alliance. Capacity Planning Framework. 2015. http://www.rehabcarealliance.ca/uploads/File/Initiatives_and_Toolkits/Definitions_and_Capacity_Planning/Capacity_Planning_Framework_FINAL.pdf. Accessed 17 Aug 2017.

[42] Pryor W, Smith F.. The rehabilitation management system: evaluating and planing physical rehabilitation services. Lyon: handicap international. 2017. http://www.hiproweb.org/uploads/tx_hidrtdocs/RMS_PG24.pdf. Accessed 12 Aug 2017.

[43] Pryor W, Newar P, Retis C, Urseau I. Compliance with standards of practice for health-related rehabilitation in low and middle-income settings: development and implementation of a novel scoring method. Disabil Rehabil. 2018:1–8. 10.1080/09638288.2018.1462409. 10.1080/09638288.2018.1462409 29663840

[44] Mannan H, McVeigh J, Amin M (2012). Core concepts of human rights and inclusion of vulnerable groups in the disability and rehabilitation policies of Malawi, Namibia, Sudan, and South Africa. J Disability Policy Studies.

[45] Center for Economic and Social Rights (CESR) (2015). The measure of Progress: how human rights should inform the sustainable development goals indicators.

[46] Meyer T, Gutenbrunner C, Kiekens C (2014). ISPRM discussion paper: proposing a conceptual description of health-related rehabilitation services. J Rehabil Med.

[47] Office of the United Nations High Commissioner for Human Rights (2010). Monitoring the Convention on the Rights of Persons with Disabilities: Guidance for human rights monitors..

[48] Office of the United Nations High Commissioner for Human Rights (2014). The Convention on the Rights of Persons with Disabilities.

[49] Skempes D, Bickenbach J (2015). Developing human rights based indicators to support country monitoring of rehabilitation services and programmes for people with disabilities: a study protocol. BMC Int Health Hum Rights.

[50] Kane M, Trochim WM. Concept mapping for planning and evaluation. Thousand Oaks: Sage Publications; 2007.

[51] van Bon-Martens MJ, van de Goor IA, van Oers HA (2017). Concept mapping as a method to enhance evidence-based public health. Eval Program Plann..

[52] Anderson LA, Slonim A (2017). Perspectives on the strategic uses of concept mapping to address public health challenges. Eval Program Plann..

[53] Rosas SR, Ridings JW (2017). The use of concept mapping in measurement development and evaluation: application and future directions. Eval Program Plann..

[54] Rosas SR (2012). The utility of concept mapping for actualizing participatory research. Cuadernos Hispanoamericanos De Psycologia.

[55] Vaughn LM, Jones JR, Booth E, Burke JG (2017). Concept mapping methodology and community-engaged research: a perfect pairing. Eval Program Plann..

[56] Velozo CA, Seel RT, Magasi S, Heinemann AW, Romero S (2012). Improving measurement methods in rehabilitation: core concepts and recommendations for scale development. Arch Phys Med Rehabil.

[57] Trochim W, Kane M (2005). Concept mapping: an introduction to structured conceptualization in health care. Int J Qual Health Care.

[58] Concept Systems Global MAX™ [program]. 2013 Version. Ithaca: Concept Systems Inc.

[59] Schiller C, Winters M, Hanson HM, Ashe MC (2013). A framework for stakeholder identification in concept mapping and health research: a novel process and its application to older adult mobility and the built environment. BMC Public Health.

[60] World Health Organization. English/French list of 206 nongovernmental organizations in official relations with WHO reflecting decisions of the 140th session of the executive board. 2017. http://www.who.int/about/collaborations/non-state-actors/non-state-actors-list.pdf?ua=1. Accessed 24 Apr 2017.

[61] Lowenson R, Laurell A, Hogstedt C, D' Ambruoso L, Shroff Z (2014). Participatory action research in health systems: A methods reader.

[62] Coxon APM (1999). Sorting data: collection and analysis.

[63] Kruskal JB, Wish M (1978). Multidimensional scaling.

[64] Davison ML (1983). Multidimensional scaling.

[65] Everitt B (1980). Cluster analysis.

[66] Rosas SR, Kane M (2012). Quality and rigor of the concept mapping methodology: a pooled study analysis. Eval Program Plann.

[67] World Health Organization (2007). Everybody's business--strengthening health systems to improve health outcomes: WHO's framework for action.

[68] De Savigny D, Taghreed A, editors. Systems thinking for health systems strengthening: Alliance for Health Policy and Systems Research, WHO, 2009.

[69] Smits SJ, Falconer J, Morland R, Strasser D (1997). The organizational context for medical rehabilitation services: a pre-chaos theory perspective. Top Stroke Rehabil.

[70] Blanchet K, Palmer J, Palanchowke R, Boggs D, Jama A, Girois S (2014). Advancing the application of systems thinking in health: analysing the contextual and social network factors influencing the use of sustainability indicators in a health system – a comparative study in Nepal and Somaliland. Health Re Policy and Sys.

[71] Sinclair E, Radford K, Grant M, Terry J (2014). Developing stroke-specific vocational rehabilitation: a soft systems analysis of current service provision. Disabil Rehabil.

[72] Esensoy AV, Carter MW (2015). Health system modelling for policy development and evaluation: using qualitative methods to capture the whole-system perspective. Oper Res Health Care.

[73] Lord JE, Stein MA (2008). Domestic incorporation of human rights law and the United Nations convention on the rights of persons with disabilities. Wash L Rev.

[74] World Health Organization (2017). Advancing the right to health: the vital role of law.

[75] McGowan AK, Lee MM, Meneses CM, Perkins J, Youdelman M (2016). Civil rights laws as tools to advance health in the twenty-first century. Annu Rev Public Health.

[76] Clarke D, Schmets G, Rajan D, Kadandale S (2016). Law, regulation and strategizing for health. Strategizing national health in the 21st century: a handbook.

[77] Sridhar D, McKee M, Ooms G (2015). Universal health coverage and the right to health from legal principle to Post-2015 indicators. Int J Health Serv.

[78] Friedman EA, Gostin LO (2015). Imagining global health with justice: in defense of the right to health. Health Care Anal.

[79] Ooms G, Brolan C, Eggermont N (2013). Universal health coverage anchored in the right to health. Bull World Health Organ.

[80] AbouZahr C, Boerma T (2005). Health information systems: the foundations of public health. Bull World Health Organ.

[81] Smith PC, Anell A, Busse R (2012). Leadership and governance in seven developed health systems. Health Policy.

[82] World Health Organization (2011). Monitoring, evaluation and review of national health strategies: a country-led platform for information and accountability.

[83] O’Neill K, Viswanathan K, Celades E, Boerma T, Schmets G, Rajan D, Kadandale S (2016). Monitoring, evaluation and review of national health policies, strategies and plans. Strategizing national health in the 21st century: a handbook.

[84] New PW, Cameron PA, Olver JH, Stoelwinder JU (2013). Defining barriers to discharge from inpatient rehabilitation, classifying their causes, and proposed performance indicators for rehabilitation patient flow. Arch Phys Med Rehabil.

[85] Mason C, Weber J, Atasoy S, Sabariego C, Cieza A (2017). Development of indicators for monitoring community-based rehabilitation. PLoS One.

[86] Keith RA (1998). Patient satisfaction and rehabilitation services. Arch Phys Med Rehabil.

[87] Mpinga EK, Chastonay P (2011). Satisfaction of patients: a right to health indicator?. Health Policy.

[88] Gupta N, Castillo-Laborde C, Landry MD. Health-related rehabilitation services: assessing the global supply of and need for human resources. BMC Health Serv Res. 2011;11. 10.1186/1472-6963-11-276. 10.1186/1472-6963-11-276 PMC3207892 22004560

[89] Backman G, Hunt P, Khosla R (2008). Health systems and the right to health: an assessment of 194 countries. Lancet.

[90] Office of the United Nations High Commissioner for Human Rights (2012). Human Rights Indicators: A Guide to Measurement and Implementation.

[91] Raub A, Latz I, Sprague A, Stein MA, Heymann J (2016). Constitutional rights of persons with disabilities: an analysis of 193 National Constitutions. Harv Hum Rights J.

[92] MacLachlan M, Scherer MJ (2018). Systems thinking for assistive technology: a commentary on the GREAT summit. Disabil Rehabil Assist Technol.

[93] MacLachlan M, Banes D, Bell D (2018). Assistive technology policy: a position paper from the first global research, innovation, and education on assistive technology (GREAT) summit. Disabil Rehabil Assist Technol.

[94] Borg J, Larsson S, Östergren PO (2011). The right to assistive technology: for whom, for what, and by whom?. Disabil Soc.

[95] Tebbutt E, Brodmann R, Borg J, MacLachlan M, Khasnabis C, Horvath R (2016). Assistive products and the sustainable development goals (SDGs). Glob Health.

[96] Hosseinpoor AR, Bergen N, Koller T (2014). Equity-oriented monitoring in the context of universal health coverage. PLoS Med.

[97] Swanson RC, Atun R, Best A (2015). Strengthening health systems in low-income countries by enhancing organizational capacities and improving institutions. Glob Health.

[98] Tomlinson M, Swartz L, Officer A, Chan KY, Rudan I, Saxena S (2009). Research priorities for health of people with disabilities: an expert opinion exercise. Lancet.

[99] Darzi AJ, Officer A, Abualghaib O, Akl EA (2016). Stakeholders’ perceptions of rehabilitation services for individuals living with disability: a survey study. Health Qual Life Outcomes.

[100] Allin S, Masseria C (2009). Unmet need as an indicator of health care access. Euro Health.

[101] MacLachlan M, Mannan H, McAuliffe E (2011). Access to health care of persons with disabilities as an indicator of equity in health systems. Open Med.

[102] Klassen A, Miller A, Anderson N, Shen J, Schiariti V, O'Donnell M (2010). Performance measurement and improvement frameworks in health, education and social services systems: a systematic review. Int J Qual Health Care.

[103] OECD (2017). Recommendations to OECD ministers of health from the high level reflection group on the future of health statistics.

[104] Townsend C, Whiteford H, Baingana F (2004). The mental health policy template: domains and elements for mental health policy formulation. Int Rev Psychiatry.

[105] Saxena S, Lora A, Van Ommeren M, Barrett T, Morris J, Saraceno B (2007). WHO's assessment instrument for mental health systems: collecting essential information for policy and service delivery. Psychiatr Serv.

[106] Funk M, Freeman M (2011). Framework and methodology for evaluating mental health policy and plans. Int J Health Plann Manag.

[107] Disabled People’s International. Position paper on CBR and WHO’s CBR Guidelines 2010, 2012. https://www.disabledpeoplesinternational.org/documents/DPI%20Position%20Paper%20on%20CBR%20Guidelines%20Final.pdf. Accessed 15 July 2018.

[108] Young B, Bagley H (2016). Including patients in core outcome set development: issues to consider based on three workshops with around 100 international delegates. Res Involv Engagem.

[109] Burke JG, O’Campo P, Peak GL, Gielen AC, McDonnell KA, Trochim WMK (2005). An introduction to concept mapping as a participatory public Health Research method. Qual Health Res.

[110] Amin M, MacLachlan M, Mannan H (2011). EquiFrame: a framework for analysis of the inclusion of human rights and vulnerable groups in health policies. Health hum. Rights.

