# Supplementary material for: Using concept mapping to develop a human rights based indicator framework to assess country efforts to strengthen rehabilitation provision and policy: the Rehabilitation System Diagnosis and Dialogue framework (RESYST)
Source: Global Health. 2018 Oct 1;14:96. doi: 10.1186/s12992-018-0410-5 (PMC6167891; doi:10.1186/s12992-018-0410-5)
Supplement: Supplementary file 1 — Indicators per cluster. Rights based indicators for rehabilitation contributed by stakeholder-participants with Bridging Index and rating scores for relative importance and feasibility to implement, arranged by cluster in descending order of importance. (DOCX 37 kb) [file 12992_2018_410_MOESM1_ESM.docx]

**Additional File 2** Rights based indicators for rehabilitation contributed by stakeholder-participants with Bridging Index and rating scores for relative importance and feasibility to implement, arranged by cluster in descending order of importance.

| No* Indicators per cluster | |  | Importance | | | Feasibility | | |
| --- | --- | --- | --- | --- | --- | --- | --- | --- |
|  | | **BI^†^** | **Mean** | **SD** | **95% CI** | **Mean** | **SD** | **95% CI** |
| **Legal Commitments and Strategic Priorities** | | **0.09** | **3.15** | **-0.87** | **(3.08; 3.22)** | **3.32** | **-0.83** | **(3.25; 3.39)** |
| 103 | Legislative provision prohibiting compulsory medical treatment and experimentation (yes/no). | 0 | 2.97 | -1.07 | (2.63; 3.32) | 3.53 | -0.7 | (3.3; 3.76) |
| 20 | Constitutional guarantees to disability equality - The State takes at least one approach to disability equality and nondiscrimination (yes/no). | 0 | 3.35 | -0.92 | (3.06; 3.65) | 3.61 | -0.69 | (3.39; 3.84) |
| 79 | State law explicitly prohibits discrimination in health insurance on the ground of disability or other pre-existing condition (yes/no). | 0.01 | 3.42 | -0.77 | (3.17; 3.66) | 3.54 | -0.78 | (3.29; 3.8) |
| 14 | Date of entry into force and coverage of domestic legislation for the implementation of the right to health of persons with disability, including legislation on rehabilitation care. | 0.01 | 3.14 | -0.79 | (2.88; 3.39) | 3.44 | -0.84 | (3.17; 3.72) |
| 63 | The State has a law to ensure universal access to comprehensive rehabilitative care and assistive products for all (yes/no). | 0.01 | 3.43 | -0.77 | (3.19; 3.68) | 3.57 | -0.7 | (3.34; 3.8) |
| 15 | The concept of disability used in health laws, policies, programmes and regulations and in the collection of relevant statistical data is in line with the human rights approach to disability and the protection of the rights of all persons with disabilities regardless of impairment (yes/no) | 0.02 | 3.28 | -0.88 | (2.99; 3.56) | 2.77 | -0.91 | (2.47; 3.07) |
| 35 | Status of ratification of international human rights treaties recognizing the right to health and their optional protocols. | 0.03 | 3.19 | -0.88 | (2.91; 3.47) | 3.67 | -0.68 | (3.45; 3.89) |
| 28 | State regulations require healthcare providers to implement policies, procedures and/or protocols for partnering with patients, carers and consumers in: (i) Strategic and operational/services planning (yes/no) (ii) Decision-making about safety and quality initiatives (yes/no) (iii) Quality improvement activities (yes/no). | 0.04 | 2.95 | -0.78 | (2.69; 3.2) | 2.78 | -1.05 | (2.44; 3.12) |
| 56 | Law requires fines for the violation of accessibility standards in healthcare facilities (yes/no). | 0.04 | 2.7 | -0.97 | (2.39; 3.01) | 3.2 | -0.99 | (2.88; 3.52) |
| 83 | Date of entry into force and coverage of the right to health of persons with disability in the constitution or other form of superior law. | 0.04 | 2.89 | -0.99 | (2.57; 3.21) | 3.41 | -0.78 | (3.16; 3.67) |
| 17 | National health or disability strategy addresses priority health related rehabilitation issues (yes/no). Describe and specify. Timeframe and coverage. | 0.09 | 3.41 | -0.76 | (3.16; 3.65) | 3.29 | -0.89 | (2.99; 3.58) |
| 66 | National disaster preparedness and relief plans are inclusive of health related rehabilitation (yes/no). | 0.13 | 3.27 | -0.8 | (3.01; 3.53) | 3.37 | -0.69 | (3.15; 3.6) |
| 50 | Evidence documenting (a) establishment of an operational, budgeted, multi sectoral national rehabilitation action plan aligned with WHO international and or regional action plans, (b) target setting process, (c) implementation activities, (d) monitoring and evaluation plan. | 0.13 | 3.41 | -0.72 | (3.17; 3.64) | 3 | -0.91 | (2.7; 3.3) |
| 81 | Legally binding national accessibility standards/guidelines established and documented (yes/no). Year of adoption. | 0.13 | 3.22 | -0.93 | (2.92; 3.52) | 3.54 | -0.66 | (3.33; 3.76) |
| 39 | The State's most recent overseas development assistance policy framework includes specific provisions to strengthen rehabilitation services and assistive technologies (yes/no). | 0.22 | 2.68 | -0.97 | (2.36; 2.99) | 3.11 | -0.99 | (2.79; 3.44) |
| 97 | Existence of an Operational Unit, Branch or Dept. in the Ministry of Health (or other Ministry) with responsibility for rehabilitation services/ assistive technologies policy development, implementation, monitoring and evaluation (yes/no). Jurisdiction and scope. | 0.28 | 3.22 | -0.79 | (2.96; 3.47) | 3.39 | -0.84 | (3.12; 3.66) |
| 88 | Existence of government approved evidence based guidelines for the rehabilitation of a wide range of disabling conditions through a multidisciplinary team approach (yes/no). | 0.29 | 3.03 | -0.83 | (2.76; 3.3) | 3.2 | -0.8 | (2.94; 3.46) |
| **Monitoring and Accountability** | | **0.49** | **3.03** | **-0.83** | **(2.93; 3.13)** | **2.83** | **-0.95** | **(2.71; 2.95)** |
| 52 | Existence of a unified accounting system to track allocation of funds to health related rehabilitation services integrated within the overall health expenditure tracking system (yes/no). | 0.4 | 2.95 | -0.88 | (2.66; 3.23) | 2.8 | -1.08 | (2.45; 3.15) |
| 58 | Existence of a national set of relevant indicators with targets and annual reporting to inform annual rehabilitation sector reviews and other planning cycles (yes/no). | 0.41 | 3.24 | -0.89 | (2.95; 3.53) | 3.14 | -0.73 | (2.9; 3.38) |
| 18 | Rehabilitation service delivery regulations, quality specifications and professional standards are established and documented (yes/no).Year of last update. | 0.45 | 3.14 | -0.75 | (2.89; 3.38) | 3.08 | -0.87 | (2.8; 3.37) |
| 48 | The State has conducted an overall assessment of the performance of the rehabilitation care system in the last 5 years (yes/no). | 0.45 | 3.24 | -0.8 | (2.99; 3.5) | 3.23 | -0.88 | (2.94; 3.52) |
| 54 | Percentage of public health campaigns including clear messages about the benefits of health related rehabilitation as % of public health campaigns run by the State in the reporting period. | 0.51 | 2.56 | -0.81 | (2.29; 2.82) | 2.46 | -0.95 | (2.15; 2.77) |
| 53 | Number of complaints on the right to health received by persons with disabilities concerning rehabilitation issues, investigated or adjudicated by the competent national human rights protection agencies in the State, and the proportion responded to effectively by the Government in the reporting period. | 0.58 | 2.86 | -0.89 | (2.58; 3.15) | 2.37 | -1.09 | (2.02; 2.73) |
| 37 | Availability of an integrated Information System on the health-related rehabilitation workforce, providing periodically updated data on the size, type, geographical distribution, competencies and skill mix of the national stock of workers. | 0.6 | 3.19 | -0.78 | (2.94; 3.44) | 2.72 | -0.97 | (2.4; 3.04) |
| **Evidence Informed and Rights Based Programming** | | **0.25** | **2.96** | **-0.86** | **(2.88; 3.04)** | **3.14** | **-0.86** | **(3.06; 3.22)** |
| 104 | The State has clearly defined priorities for research on rehabilitation in the most recent national health research policy statements (yes/no). Timeframe and coverage. | 0.08 | 2.78 | -0.89 | (2.5; 3.07) | 3.22 | -0.83 | (2.95; 3.49) |
| 40 | Existence of national multi-sectoral commission, agency or mechanism for the coordination of disability policy and the implementation of the CRPD (yes/no). Scope & functions. | 0.08 | 3.33 | -0.83 | (3.07; 3.6) | 3.51 | -0.61 | (3.31; 3.71) |
| 71 | The State has a budgeted plan to raise awareness about disability issues among health professionals which involves persons with disabilities and their representative organizations (yes/no). Timeframe and coverage. | 0.11 | 2.92 | -0.8 | (2.66; 3.18) | 3.06 | -0.76 | (2.81; 3.31) |
| 70 | The State has a systematic plan and coordinating unit for acquiring and using rehabilitation research information and for sharing and transferring knowledge (yes/no). | 0.14 | 3 | -0.78 | (2.75; 3.25) | 3.06 | -0.87 | (2.77; 3.34) |
| 100 | Existence of accessible pre-judicial mechanisms to lodge complaints alleging breach of obligations connected to the right to health. Jurisdiction and scope. | 0.19 | 2.92 | -0.83 | (2.65; 3.19) | 2.88 | -0.91 | (2.58; 3.18) |
| 59 | Confidentiality of health and rehabilitation records protected by law (yes/no). | 0.2 | 2.83 | -0.88 | (2.55; 3.12) | 3.46 | -0.82 | (3.19; 3.72) |
| 49 | The concept of "Assistive Devices' Single Window Service" provision is documented in national policy statements and implemented. | 0.22 | 2.49 | -1.02 | (2.16; 2.81) | 2.94 | -1.03 | (2.61; 3.28) |
| 44 | Existence of a government website which meets the ISO/IEC 40500:2012 standards of accessibility for web content with latest report and data about rehabilitation services available to the general public (yes/no). | 0.25 | 2.89 | -0.97 | (2.58; 3.2) | 3.43 | -0.65 | (3.21; 3.64) |
| 67 | Evidence on the existence of formal collaboration between (a) the department/agency responsible for rehabilitation and (b) the department/agency responsible for: (i) employment, (ii) education, (iii) welfare (iii) CRPD implementation. | 0.25 | 3.35 | -0.63 | (3.15; 3.56) | 2.86 | -0.97 | (2.54; 3.18) |
| 57 | State has established inclusive procedures or mechanisms for consultation with disabled people's organizations at national, sub-national and local levels (yes/no). | 0.31 | 3.35 | -0.72 | (3.12; 3.58) | 3.15 | -0.82 | (2.88; 3.42) |
| 24 | Charter of patient rights published and available in accessible formats (yes/no). | 0.31 | 3.08 | -0.89 | (2.79; 3.37) | 3.46 | -0.74 | (3.21; 3.7) |
| 75 | Existence of a participatory forum and disability inclusive process to coordinate the setting of national rehabilitation research priorities (yes/no). | 0.55 | 2.95 | -0.94 | (2.64; 3.25) | 3.14 | -0.94 | (2.83; 3.45) |
| 85 | Government Research and Development (R&D) expenditure directed to rehabilitation sciences and engineering as % of gross domestic product (GDP). | 0.55 | 2.54 | -0.9 | (2.25; 2.83) | 2.69 | -1.04 | (2.36; 3.03) |
| **Workforce Development** | | **0.76** | **2.91** | **-0.87** | **(2.78; 3.04)** | **3.13** | **-0.82** | **(3.01; 3.25)** |
| 1 | The State's workforce policies or programmes include a plan for national self-sufficiency for rehabilitation professionals and document incentives to promote retention of rehab professionals (yes/no). | 0.58 | 2.86 | -1.03 | (2.53; 3.2) | 3.19 | -0.86 | (2.91; 3.47) |
| 19 | Existence of disability human rights education as an element of the accreditation standards used at the national level in the field of rehabilitation. | 0.75 | 3.03 | -0.76 | (2.78; 3.27) | 3.11 | -0.75 | (2.87; 3.36) |
| 69 | Existence of human resources for health unit that is responsible for developing and monitoring policies and plans on rehabilitation workforce and negotiating intersectoral relationships with other line ministries and stakeholders (yes/no). | 0.76 | 2.89 | -0.94 | (2.59; 3.19) | 2.71 | -0.96 | (2.4; 3.03) |
| 51 | Availability of ethical standards of care for rehabilitation physicians and allied health professionals (yes/no). | 0.8 | 3 | -0.78 | (2.75; 3.25) | 3.34 | -0.73 | (3.11; 3.58) |
| 95 | Existence of a government approved curriculum for training of informal care workers in Community Based Rehabilitation (yes/no). | 0.89 | 2.78 | -0.83 | (2.51; 3.05) | 3.31 | -0.79 | (3.05; 3.56) |
| **Access Barriers** | | **0.36** | **2.89** | **-0.86** | **(2.81; 2.97)** | **2.39** | **-0.86** | **(2.31; 2.47)** |
| 101 | Timely access to rehabilitation 3 – Percentage (%) of population aged 16 or older who reported that in the last 12 months they were able to see a rehabilitation professional on the same day or next day, when needed. | 0.3 | 2.78 | -0.98 | (2.47; 3.1) | 2.14 | -0.96 | (1.83; 2.45) |
| 78 | Proportion of the population living within four hours travel to a rehabilitation/assistive technology service. (Allows for visiting a service within a day.) | 0.33 | 2.92 | -0.98 | (2.6; 3.24) | 2.4 | -0.88 | (2.11; 2.69) |
| 55 | Timely access to rehabilitation 2 - Time (median waiting time in days) between: (i) acute hospital admission until referral for rehabilitation, (ii) referral until assessment, (iii) acceptance by post-acute rehabilitation care and ready for transfer until admission. | 0.35 | 2.97 | -0.93 | (2.67; 3.27) | 2.29 | -0.93 | (1.98; 2.59) |
| 46 | Barriers to access to medical rehabilitation (%) - Reported number of persons with disabilities not having access to medical rehabilitation services due to transportation barriers, physical/geographical access barriers, waiting time, lack of information; lack of time; inadequate skills of service provider; cost or other. | 0.35 | 3.64 | -0.59 | (3.45; 3.83) | 2.34 | -0.84 | (2.07; 2.62) |
| 26 | Inequality in access to rehabilitation - Absolute difference in unmet needs for rehabilitation between people with and without clinical impairments/disabilities (trends). | 0.35 | 3 | -0.94 | (2.7; 3.3) | 2.11 | -0.92 | (1.81; 2.41) |
| 87 | National average proportion of "rehab ready" referrals (by diagnostic category) admitted to outpatient/ambulatory rehabilitative care programme | 0.35 | 2.3 | -0.74 | (2.06; 2.54) | 1.94 | -0.73 | (1.71; 2.18) |
| 16 | Rehabilitation denial rate - Number of "rehabilitation ready" referrals (by diagnostic category) declined without proper clinical justification as % of total rehabilitation ready referrals in the reporting period. | 0.35 | 2.7 | -0.91 | (2.41; 3) | 1.86 | -0.77 | (1.6; 2.11) |
| 107 | Needs for assistive products met (%) - Reported number of persons with disability using an assistive product that fits their functional needs | 0.36 | 3.06 | -0.75 | (2.81; 3.3) | 2.37 | -0.91 | (2.07; 2.67) |
| 92 | Percentage (%) of health facilities providing medical rehabilitation services | 0.38 | 3.08 | -0.76 | (2.84; 3.33) | 3.22 | -0.68 | (3; 3.44) |
| 74 | Timely access to rehabilitation 1 - Percentage of acute hospital length of stay spent waiting for a post-acute rehabilitation bed | 0.38 | 2.78 | -0.95 | (2.48; 3.09) | 2.26 | -0.92 | (1.96; 2.56) |
| 60 | Number of licensed assistive products dispensers by geographic region. | 0.38 | 2.49 | -0.8 | (2.23; 2.75) | 2.86 | -0.85 | (2.58; 3.13) |
| 82 | AT affordability - Percentage (%) of the per capita GDP or income required to purchase a wheelchair (average price). | 0.43 | 2.92 | -0.87 | (2.63; 3.2) | 2.94 | -0.87 | (2.66; 3.23) |
| **Service Coverage, Utilization and Outcomes** | | **0.37** | **2.87** | **-0.85** | **(2.8; 2.94)** | **2.6** | **-0.85** | **(2.53; 2.67)** |
| 8 | Number of outpatient rehabilitation visits per capita per year. | 0.28 | 2.57 | -0.83 | (2.3; 2.84) | 3.17 | -0.88 | (2.88; 3.45) |
| 102 | Median number of patient served per rehabilitation programme per year. | 0.29 | 2.49 | -0.8 | (2.23; 2.75) | 2.69 | -0.82 | (2.43; 2.96) |
| 34 | Inpatient rehabilitation care beds/population ratio. | 0.32 | 2.78 | -0.95 | (2.48; 3.09) | 3.36 | -0.87 | (3.08; 3.64) |
| 61 | Patient status at discharge - National average percentage (%) of rehabilitation inpatients with improved function scores at discharge (compared with scores measured at admission). | 0.33 | 2.92 | -0.97 | (2.6; 3.23) | 2.34 | -0.76 | (2.09; 2.59) |
| 65 | Unmet needs for medical rehabilitation (%) - Reported number of persons with disability that needed medical rehabilitation services or assistive devices in the last 12 months and did not get the services they need, stratified by age, income, geographic region and educational level | 0.34 | 3.46 | -0.77 | (3.21; 3.71) | 2.26 | -0.79 | (2.01; 2.52) |
| 47 | Discharge destinations (selected tracer conditions) in the reporting period. | 0.34 | 2.3 | -0.88 | (2.01; 2.58) | 2.26 | -0.95 | (1.95; 2.57) |
| 12 | Rehabilitation hospital admission/discharge rates - Number of rehabilitation unit or hospital admissions/discharges per 100 000 (by diagnostic category). | 0.35 | 2.68 | -0.82 | (2.41; 2.94) | 3.17 | -0.77 | (2.91; 3.42) |
| 62 | Patient satisfaction with rehabilitation provider. | 0.39 | 2.7 | -0.85 | (2.43; 2.98) | 2.34 | -0.76 | (2.09; 2.59) |
| 72 | Patient status after discharge - National average percentage of discharged rehabilitation inpatients who still live in the community 90 days post discharge. | 0.39 | 2.59 | -0.96 | (2.29; 2.9) | 2.06 | -0.91 | (1.76; 2.35) |
| 10 | Number of multidisciplinary rehabilitation programmes per 1 000 000 - (e.g., cardiac, cancer, stroke, spinal cord injury, paediatric rehabilitation programmes). | 0.39 | 3.16 | -0.76 | (2.92; 3.41) | 3 | -0.86 | (2.72; 3.28) |
| 29 | Proportion of persons with disability living in complex emergency environments that can access comprehensive rehabilitation services | 0.41 | 2.92 | -0.89 | (2.63; 3.21) | 2.19 | -0.92 | (1.89; 2.5) |
| 7 | Number of Community Based Rehabilitation providers/population ratio (per 100 000) | 0.41 | 3.27 | -0.69 | (3.05; 3.49) | 3.06 | -0.83 | (2.79; 3.33) |
| 96 | Financial barriers to access to rehabilitation (%) - Reported number of persons with disability that have forgone prescribed rehabilitation treatment due to financial reasons in the last 12 months, disaggregated by income level, sex and age | 0.42 | 3.19 | -0.84 | (2.92; 3.46) | 2.19 | -0.95 | (1.88; 2.51) |
| 31 | Financial barriers to AT (%) - Reported number of persons with disability who didn't get their prescribed assistive devices because of their cost | 0.57 | 3.22 | -0.8 | (2.97; 3.48) | 2.37 | -0.84 | (2.1; 2.65) |
| **Service Financing and Quality Control** | | **0.58** | **2.82** | **-0.85** | **(2.75; 2.89)** | **2.79** | **-0.83** | **(2.72; 2.86)** |
| 38 | Percent (%) of healthcare organizations providing medical rehabilitation who currently have processes to involve community/client input for planning and performance monitoring of the organization's services (e.g. advisory committees, focus groups). | 0.41 | 2.84 | -0.87 | (2.56; 3.12) | 2.44 | -0.88 | (2.16; 2.73) |
| 77 | Percentage (%) of inpatient rehabilitation facility discharges for which a discharge report is made available to primary care. | 0.46 | 2.35 | -0.89 | (2.06; 2.64) | 2.34 | -0.94 | (2.04; 2.65) |
| 106 | Percentage (%) of Net Official Development Assistance (ODA) for health (provided or received by the State) directed to the health related rehabilitation sector. | 0.48 | 2.65 | -0.86 | (2.37; 2.92) | 2.85 | -0.82 | (2.58; 3.12) |
| 90 | Evidence of economic strategies to incentivize manufacture and/or provision/distribution of assistive products. | 0.49 | 2.65 | -0.95 | (2.34; 2.95) | 2.63 | -0.97 | (2.31; 2.95) |
| 21 | Evidence (including of qualitative nature) of gender sensitiveness of rehabilitation services. | 0.51 | 2.95 | -0.85 | (2.67; 3.22) | 2.42 | -0.87 | (2.13; 2.7) |
| 93 | Proportion of health facilities providing medical rehabilitation that are accredited by a recognized accreditation body. | 0.52 | 2.86 | -0.79 | (2.61; 3.12) | 3.22 | -0.76 | (2.97; 3.47) |
| 33 | A comprehensive array of medical rehabilitation services is enlisted in the State's essential health benefits package including for the purpose of maintaining current levels of functioning (yes/no). Describe and specify. | 0.52 | 3.33 | -0.83 | (3.07; 3.6) | 3.31 | -0.82 | (3.04; 3.57) |
| 73 | Expenditure trends on (i) rehabilitation care (inpatient, outpatient and community based) as % of government health expenditure (ii) assistive products as % of government health expenditure. | 0.61 | 3 | -0.82 | (2.74; 3.26) | 2.77 | -0.81 | (2.51; 3.04) |
| 22 | Information about the household catastrophic/impoverishing expenditure on rehabilitation care is available and regularly updated (yes/no). | 0.61 | 2.65 | -0.89 | (2.36; 2.94) | 2.14 | -0.99 | (1.82; 2.46) |
| 9 | Percentage (%) of health facilities/units offering medical rehabilitation with established quality improvement teams, by facility type. | 0.62 | 2.97 | -0.9 | (2.68; 3.26) | 2.83 | -0.66 | (2.61; 3.05) |
| 11 | Percentage (%) of WHO recommended priority assistive products included in the national assistive products list for procurement and reimbursement. | 0.68 | 3.3 | -0.78 | (3.05; 3.55) | 3.54 | -0.66 | (3.33; 3.76) |
| 36 | The State subsidizes disabled people’s travel costs to access rehabilitation services that are not available near their place of residence. | 0.71 | 2.95 | -0.85 | (2.67; 3.22) | 3.03 | -0.77 | (2.77; 3.28) |
| 2 | Percentage (%) of rehabilitation facilities with confidentiality protocols on health and rehabilitation information. | 0.72 | 2.43 | -0.87 | (2.15; 2.71) | 2.97 | -0.7 | (2.74; 3.2) |
| 3 | Proportion of facilities providing medical rehabilitation with at least one yearly external review/inspection of human rights protection of patients, by type of facility. | 0.76 | 2.59 | -0.83 | (2.33; 2.86) | 2.54 | -0.82 | (2.28; 2.81) |
| **Higher Education** | | **0.66** | **2.82** | **-0.85** | **(2.68; 2.96)** | **3.11** | **-0.88** | **(2.97; 3.25)** |
| 94 | Percent (%) of undergraduate healthcare professions' curricula that include a module on disability ethics/human rights, by specialization. | 0.63 | 2.81 | -0.84 | (2.54; 3.08) | 3.06 | -0.92 | (2.75; 3.36) |
| 45 | Number of graduates from rehabilitation education and training programmes during the last academic year by cadre, per 1000 population. | 0.63 | 2.76 | -0.8 | (2.5; 3.01) | 2.94 | -0.94 | (2.64; 3.25) |
| 80 | Number of postgraduate programmes in rehabilitation sciences and related fields available to allied health professionals by occupation/specialization recognized by their respective licensing bodies or equivalent health professions/education council. | 0.67 | 2.68 | -0.91 | (2.38; 2.97) | 3.26 | -0.75 | (3.02; 3.51) |
| 25 | Training in physical medicine and rehabilitation available for doctors. This refers to a residency programme in Physical Medicine and Rehabilitation (PMR) or specialist certification in PRM which is recognized by the medical council or the equivalent licensing body of the country (yes/no). | 0.71 | 3.03 | -0.83 | (2.76; 3.3) | 3.19 | -0.89 | (2.9; 3.48) |
| **Workforce Planning and Performance** | | **0.77** | **2.76** | **-0.85** | **(2.68; 2.84)** | **2.64** | **-0.84** | **(2.56; 2.72)** |
| 43 | Rehabilitation professionals / rehabilitation beds ratio. | 0.54 | 2.54 | -0.82 | (2.28; 2.81) | 3.09 | -0.89 | (2.8; 3.38) |
| 41 | Number of entrants into Community Based Rehabilitation training programmes (with a nationally approved curriculum) per year. | 0.67 | 2.7 | -0.74 | (2.46; 2.94) | 2.8 | -0.96 | (2.49; 3.11) |
| 27 | Specialists in Rehabilitation Medicine / physicians ratio. | 0.69 | 2.81 | -0.92 | (2.51; 3.1) | 3.37 | -0.73 | (3.13; 3.61) |
| 99 | Percent of registered nurses with rehabilitation certification. | 0.71 | 2.38 | -0.83 | (2.11; 2.65) | 3.25 | -0.69 | (3.02; 3.48) |
| 76 | Total number of rehabilitation workers employed in hospitals. | 0.72 | 2.68 | -0.94 | (2.37; 2.98) | 3.06 | -0.87 | (2.77; 3.34) |
| 64 | Percentage (%) of persons with disability that feel they have received sufficient information and been sufficiently involved in making decisions about their rehabilitation treatment compared to people without disability | 0.72 | 2.95 | -0.91 | (2.65; 3.24) | 2.26 | -0.89 | (1.97; 2.55) |
| 98 | Proportion of organizations providing medical rehabilitation with at least one day training, meeting or other type of working session on human rights protection of persons with disabilities in the last two years. Report total number of beneficiaries/participants by occupational level/specialization. | 0.73 | 2.35 | -0.86 | (2.08; 2.63) | 2.11 | -0.75 | (1.87; 2.36) |
| 86 | Rehabilitation workforce density by occupation/specialization and activity level. | 0.75 | 2.89 | -0.84 | (2.62; 3.16) | 2.58 | -0.87 | (2.3; 2.87) |
| 84 | Assistive devices training needs met – Percentage (%) of persons with disability using assistive devices that know how to maintain them. | 0.81 | 2.86 | -0.76 | (2.62; 3.11) | 2.11 | -0.9 | (1.82; 2.41) |
| 91 | Percentage of rehabilitation service users who said they have been sufficiently involved in decisions about their care as much as they wanted to be. | 0.88 | 2.89 | -0.82 | (2.62; 3.15) | 2.19 | -0.89 | (1.9; 2.48) |
| 13 | Self-perceived community integration – Percentage (%) of survey respondents with disability who would rate their level of community integration as "7"out of "10" or higher. | 0.98 | 3.03 | -0.96 | (2.72; 3.34) | 2.5 | -0.85 | (2.22; 2.78) |
| 4 | Percentage (%) of persons with disability reporting having personally felt discriminated against or harassed during rehabilitation within the last 12 months on the basis of a ground of discrimination prohibited under international human rights law compared to people without disability. | 1 | 3.03 | -0.8 | (2.77; 3.28) | 2.33 | -0.72 | (2.1; 2.57) |
| **Disability Statistics** | | **0.47** | **2.74** | **-1.06** | **(2.59; 2.89)** | **2.99** | **-0.91** | **(2.86; 3.12)** |
| 89 | New invalidity / disability cases per 100 000, by sex and age. | 0.41 | 2.49 | -1.12 | (2.13; 2.85) | 2.69 | -0.93 | (2.38; 2.99) |
| 23 | Standardized Mortality Ratios (SMRs) of people with disability (selected tracer conditions) compared with the general population. | 0.46 | 2.64 | -0.99 | (2.32; 2.96) | 2.61 | -0.99 | (2.29; 2.94) |
| 42 | People receiving disability benefits per 100 000. | 0.47 | 2.59 | -0.98 | (2.28; 2.91) | 3.57 | -0.7 | (3.34; 3.8) |
| 5 | Return to work rates - Average national percentage of vocational rehabilitation clients of working age who are engaged in sustainable employment 3-6 months after closure and were employed before entering vocational rehabilitation. | 0.49 | 3.11 | -1.02 | (2.78; 3.44) | 2.86 | -1 | (2.53; 3.19) |
| 6 | Disability prevalence. | 0.51 | 2.86 | -1.16 | (2.49; 3.24) | 3.22 | -0.87 | (2.94; 3.5) |
| **Social Mobilization and Research** | | **0.57** | **2.23** | **-0.85** | **(2.09; 2.37)** | **2.43** | **-0.94** | **(2.28; 2.58)** |
| 68 | Socio-cultural environment of rehabilitation services: beliefs and cultural attitudes of health workers, care givers and their stakeholders which either prevents or enhance access to rehabilitation services. Describe and specify. | 0.52 | 2.7 | -0.85 | (2.43; 2.98) | 1.91 | -0.82 | (1.65; 2.18) |
| 30 | Number of registered and/or active NGOs (per 100 000 persons) involved in rehabilitation advocacy. | 0.52 | 2.41 | -0.9 | (2.12; 2.69) | 2.67 | -0.86 | (2.39; 2.95) |
| 32 | Number of patent applications filed in the assistive technology sector under the PCT as % of total number of patent applications filed in the reporting period. | 0.55 | 1.89 | -0.88 | (1.61; 2.17) | 2.6 | -1.03 | (2.26; 2.94) |
| 105 | Number of registered prospective randomized trials (clinical/non clinical) in rehabilitation as a % of all randomized trials registered in the reporting period. | 0.68 | 1.92 | -0.8 | (1.66; 2.18) | 2.54 | -1.01 | (2.21; 2.87) |

* Number corresponds to the number that was randomly assigned to the indicator after the brainstorming phase.

† Indicators within each cluster are arranged by descending order of Bridging Index (BI) score. BI is a measure of the internal cohesiveness of the cluster. Values range from zero to one (0-1). Clusters with low BI are internally more cohesive as the indicators contained therein were more frequently sorted together by participants. Clusters with higher BI contain broader concepts and are highly likely to be related to other domains on the concept map thus showing the degree of interconnectedness of the cluster with other parts of the framework .

Cluster rating scores are based on the mean rating for all indicators within the cluster.
