# Supplementary material for: Using concept mapping to develop a human rights based indicator framework to assess country efforts to strengthen rehabilitation provision and policy: the Rehabilitation System Diagnosis and Dialogue framework (RESYST)
Source: Global Health. 2018 Oct 1;14:96. doi: 10.1186/s12992-018-0410-5 (PMC6167891; doi:10.1186/s12992-018-0410-5)
Supplement: Supplementary file 2 — Stakeholder panel membership. Organizational affiliations of participants in the sorting and rating phase. (DOCX 77 kb) [file 12992_2018_410_MOESM2_ESM.docx]

**Additional File 1. Stakeholder panel membership**

| No | Organization | Type |
| --- | --- | --- |
| 1. | Neurological Institute Carlo Besta (IRCCS), Italy | Research |
| 2. | Ministry of Social Development, New Zealand | Government/Political |
| 3. | Rehabilitation International | DPO |
| 4. | Swiss Paraplegic Center, Switzerland | Service Provider |
| 5. | SINTEF, Norway | Research |
| 6. | Nossal Institute of Global Health, University of Melbourne, Australia | Research |
| 7. | WHO Collaboration Centre for the Family of International Classifications | Research |
| 8. | Handicap International | NGO |
| 9. | World Confederation of Physical Therapy, UK | Professional |
| 10. | UN Washington Group on Disability Statistics, USA | Research |
| 11. | Leonard Cheshire Centre on Disability, University College London, UK | Research |
| 12. | Anglo-Nigerian Welfare Association for the Blind (ANWAB), Nigeria | DPO |
| 13. | UN Committee on the Rights of Persons with Disabilities | Government/Political |
| 14. | Department of Rehabilitation Medicine, PNS Shifa Hospital, DHA II, Karachi , Pakistan | Research/  Service Provider |
| 15. | Ministry of Health, Republic of Seychelles | Government/Political |
| 16. | Pakistan Disabled People's Organisation, Pakistan | DPO |
| 17. | CBR Network Asia Pacific | DPO |
| 18. | University of Sydney, Centre for Disability Research and Policy, Australia | Research |
| 19. | WHO Collaborating Centre in Health Workforce Development in Rehabilitation and Long Term Care, Australia | Research |
| 20. | St. Petersburg Scientific and Practical Centre of Medical and Social Expertise, Prosthetics and Rehabilitation named after G.A. Albrecht” of the Ministry of Labour and Social Protection of the Russian Federation, Russia | Research/  Service Provider |
| 21. | Enablement, Netherlands |  |
| 22. | International Centre for Evidence on Disability, London School of Hygiene and Tropical Medicine, LSHTM, UK | Research |
| 23. | University of Rochester Medical Center, USA, Center on Effective Rehabilitation Technology (CERT), USA | Research |
| 24. | International Association of Anti-Leprosy Associations | DPO |
| 25. | Burton Blatt Institute (BBI) at Syracuse University College of Law, USA | Research |
| 26. | Harvard Law Project on Disability, Harvard University, USA | Research |
| 27. | University of Cape Town, South Africa | Research |
| 28. | CBM USA | NGO |
| 29. | CBR Advisory Working Group, CBM International, Germany | NGO |
| 30. | WHO Collaborating Centre for Health Workforce Policy and Planning, Institute of Hygiene and Tropical Medicine - NOVA University of Lisbon (IHMT-UNL), Portugal | Research |
| 31. | WHO Collaborating Center for Rehabilitation, China | Research |
| 32. | Abilia Foundation | NGO |
| 33. | Thomas Jefferson University, USA | Research |
| 34. | International Society of Physical and Rehabilitation Medicine (ISPRM) | Professional |
| 35. | University of Maastricht Law School, Netherlands | Research |
| 36. | University of East Anglia Norwich Medical School, UK | Research |
| 37. | University of Munich, Germany | Research |
| 38. | Duke University, USA | Research |
| 39. | WHO Country Office Tajikistan | Government/Political |
| 40. | Fordham University, USA | Research |
| 41. | Sichuan University and Hong Kong Polytechnic University, Sichuan, Chengdu, China | Research |
| 42. | Department of Rehabilitation Medicine, University Medical Center Utrecht, Netherlands | Research/  Service Provider |
| 43. | WHO Global Cooperation on Assistive Technology - Prosthetics and Orthotics Service Standards Committee, Switzerland | Research  Government/Political |
| 44. | Department of Human & Social Sciences and International Relations, National School of Public Health, France | Research |
| 45. | MoveAbility Foundation - International Committee of the Red Cross (ICRC), Lome, Tongo | NGO |
| 47. | Dept. of Rehabilitation Medicine, University of Malaysia, Malaysia | Research |
